# Adolescents with self-injurious behavior in emergency services: a look at comprehensive care

**DOI:** 10.1590/1980-220X-REEUSP-2025-0030en

**Published:** 2025-08-11

**Authors:** Janiely Aparecida Senne de Sousa Leite, Ana Paula Miranda de Araújo Soares, Nathalia Vitória de Carvalho Martinez, Aline Conceição Silva, Angelina Lettiere-Viana, Tauani Zampieri Fermino, Diene Monique Carlos

**Affiliations:** 1 Universidade Federal de São Carlos, Departamento de Enfermagem, São Carlos, SP, Brazil.; 2 Universidade de São Paulo, Escola de Enfermagem de Ribeirão Preto, Ribeirão Preto, SP, Brazil.; 3 Universidade de São Paulo, Escola de Enfermagem, São Paulo, SP, Brazil.

**Keywords:** Adolescent, Self-Injurious Behavior, Nursing, Emergencies, Qualitative Research

## Abstract

**Objective:**

To understand the care provided to adolescents with a history of self-injury from the perspective of healthcare professionals working in emergency services.

**Method:**

Qualitative research, anchored in the concept of comprehensive care. Eight healthcare professionals working in emergency services participated, recruited using the snowball technique. Data collection took place between 2021 and 2022, based on semi-structured interviews on virtual platforms, and the data were analyzed using reflective thematic analysis.

**Results:**

Two final topics emerged: 1. Meeting the adolescent who self-injures – related feelings; and 2. Beyond that momentary injury – the organization of practices and services for the care of adolescents who self-injured.

**Conclusion:**

There are challenges related to the way of working in these settings and training weaknesses in the areas of mental health and adolescence. Networking is difficult, and care is based on the logic of referral. The concept of comprehensiveness can trigger the organization of actions, services and policies aimed at this phenomenon.

## INTRODUCTION

Adolescents have become vulnerable populations to self- inflicted violence, with impacts on the morbidity and mortality profiles of this population^(1)^. Non-suicidal self-injury (NSSI) has become a prominent phenomenon. It is defined as deliberate injuries that alter or destroy body tissue and are not socially accepted. Initially, there is no conscious intention to commit suicide, but they may be risk factors for this condition^(2)^.

Statistics have shown prevalence rates of 11.5% to 33.8% of NSSI in countries of the Global South^(3)^. In Brazil, despite the limitations of statistical data related to this phenomenon, there has been a greater incidence of self-injurious behavior in recent years^(4)^. The main method used is cutting the arms and other parts of the body, and it can also appear as burns, especially in male adolescents. It can be related to psychological distress such as depression, anxiety, and social phobia^(2,3,4,5)^.

Among the main risks of this behavior in adolescents is the difficulty regulating emotions, such as anger, anxiety and fear. The unique process of neurodevelopment in adolescence brings greater emotional responsiveness with less control and perception of risks. Other vulnerability factors identified in the literature are being female, suffering violence, conflicts in affective-sexual and family relationships, contact with peers who also self-injury, showing low self-esteem, difficulty expressing emotions, self-criticism, and irregular sleep patterns^(2,5)^.

The literature has been highlighting the importance of appropriate clinical management of NSSI, especially at the first visit, such as in emergency services (ESs). This aspect is crucial to reduce morbidity and mortality in the adolescent and young population. Despite this, care for NSSI and the availability of resources remain poorly planned and articulated, possibly due to the lack of preventive strategies or resources to identify structural elements related to NSSI in adolescence^(3)^. Young people report the need for sensitive services and compassionate care that see them as people beyond their diagnoses, making them feel safe, welcomed and supported^(6)^.

Despite the constant and growing presence of care aimed at adolescents with NSSI in ESs, scientific production is lacking, especially in countries of the Global South, signaling challenges present in this setting^(7,8)^. A study carried out with nurses in Denmark revealed the perception of managing NSSI as exhausting, endless and frustrating work, especially in psychosocial aspects^(7)^. Another Australian study demonstrated the need for continuing education aimed at building therapeutic relationships in this care^(8)^.

There is a dialogue between the health needs experienced by adolescents in situations of NSSI and the concept of comprehensiveness. As one of the fundamental principles provided for in the Brazilian Health System (In Portuguese, *Sistema Único de Saúde* – SUS), it focuses on the expanded understanding of needs and consequent adaptation of contextually articulated offers to the encounter between a person seeking the service and the health team. It has meanings related to characteristics of policies that focus on the articulation of preventive and care actions, and to health practices that are carried out in services and meetings^(9)^. It seeks to guarantee access and continuity of care at different points in Healthcare Networks (In Portuguese*, Redes de Atenção à Saúde* – RAS) through care transition. RAS are organizational models for the SUS, aiming to articulate and integrate healthcare services, including the Emergency Care Network (In Portuguese, *Rede de Atenção às Urgências e Emergências* – RAUE)^(10)^ and the Psychosocial Care Network (In Portuguese, *Rede de Atenção Psicossocial* – RAPS)^(11)^, which are related to care for NSSI.

Given the above, this study aimed to understand the care provided to adolescents with a history of self-injury from the perspective of healthcare professionals working in ESs. It is understood that this object of study, aligned with nursing’s perspective as a social practice, can allow advances in the construction of scientific, practical and managerial knowledge in a field that is still timidly occupied by the area’s agenda.

## METHOD

### Study Design

This is qualitative research^(12)^ anchored in the concept of comprehensive care^(9)^.

### Population and Selection Criteria

Snowball sampling was used to locate people with the profile required for the research, using key informants, named as seeds. Thus, the seeds helped the researcher to initiate contacts and to feel out the group to be researched. Then, the people indicated by the seeds were asked to indicate new contacts with the desired characteristics, from their own personal network and so on so that the sampling frame could grow with each interview^(13)^. The first seed was a member of the lead author’s research group.

Healthcare professionals with higher education who had already worked in care for adolescents with a history of self- injury, especially with experience in emergency units at any level of SUS healthcare or in supplementary (private) health institutions, and who had worked in the services for at least six months were included. Professionals who were not actively working in the services during the data collection period and who did not respond after three contact attempts were excluded.

Throughout the process, 23 people were contacted to participate, five of whom refused due to the study topic; seven did not respond to attempts to contact them; and three refused due to lack of time. Thus, eight participants comprised this study.

### Data Collection

The semi-structured interview^(12)^ was used as a data collection technique. Participants were characterized using a sociodemographic questionnaire. The interviews were conducted remotely, due to the COVID-19 pandemic, and the script addressed perceptions about practices, feelings, and challenges in caring for NSSI among adolescents. In this regard, options mediated by free virtual platforms were given as participants felt more comfortable. The Informed Consent Form was sent through Google Forms®. The interviews were conducted via video call via the WhatsApp® application and lasted an average of 25 minutes. Participants were identified with the letter P and numbered in the sequence in which the interviews were conducted.

Data collection period took place between December 2021 and July 2022. In this study, we chose to seek meaning saturation, which corresponds to a deeper, more detailed and complex discussion with the data to ensure the understanding of a phenomenon of interest^(14)^.

### Data Analysis

The data were analyzed using reflective thematic analysis^(15)^. This is an analysis with inductive logic, based on data, considered a reflective, creative, subjective and deliberative process. It brings a systematic and rigorous approach to coding and creating topics that is both fluid and recursive. The following stages were followed: (I) familiarization with the data; (II) coding; (III) search for topics; (IV) topic review; (V) topic definition and naming; (VI) final writing.

### Ethical Aspects

The study followed the recommendations of Resolution 466/2012 on research involving human beings. It was initially approved by municipal and local service leaders, and was approved by the *Universidade Federal de São Carlos* Research Ethics Committee on August 22, 2019, under Certificate of Presentation for Ethical Consideration 17176219.6.0000.5504.

## RESULTS

Eight healthcare professionals participated: two men and six women. Six professionals were between 25 and 30 years old, and two were between 35 and 39 years old. Four were married and four were single. Seven had no children and one participant had a 4-year-old son. Five declared themselves to be white, one was yellow, and two were brown. Two did not belong to any religion; one was a spiritualist; and the others were Catholic. Professional training consisted of five nurses, one with a specialization and master’s degree, and three physicians (residency in otorhinolaryngology, psychiatry, and pediatrics). The length of service ranged from six months to 11 years.

Four professionals had contact with the topic of NSSI during their professional training. Three participated in updates on the topic during their professional careers. Seven professionals reported that the topic of adolescence was addressed during their undergraduate studies.

The data from the semi-structured interviews will be presented in the final topics below.

1. Meeting the adolescent who self-injures related feelings

This category permeated professionals’ understanding when meeting adolescents in ESs. Professionals showed that there is a certain trivialization of care for NSSI in adolescents, exposing preconceived ideas about the situation, characterizing it as a deliberate search for attention and understanding of taking up other people’s space:

[...] *people are a bit prejudiced, most of them. They think that “wow” the person is trying to get attention in some way, especially when it is an injury that does not pose a risk to life, so the person is trying to get attention*. (P1–Nursing)

*Unfortunately, many professionals who deal with these cases come as the adolescent, the patient wanting to get attention, etc.* (P2–Nursing)

One participant spoke about the difficulty professionals have in being in touch with NSSI and, from there, trying to understand adolescents’ experiences, thinking about care comprehensiveness. The need to develop attitudinal skills that allow being in touch with others’ pain without disregarding the impact of these phenomena on professionals’ mental health was understood:

*It’s hard to understand what adolescents are going through*. […] *it’s also challenging because, within the team, we have many people from different cultures and who engage in self- injury; it’s something violent, it’s something shocking, it’s something that causes a certain fear in people* […] *I’ve seen this happen. I’ve seen it sometimes in teams that are less trained for this type of situation, where there’s a fear, a question of stigma surrounding self-injury.* (P3–Medicine)

Actions, when faced with situations of NSSI brought by participants, can be permeated by aggressive and reckless attitudes, due to the stigmatization present, as described in the following statements:

[…] *some people are a little prejudiced and end up treating the person in a more aggressive, but arrogant way, knowing that the person caused their own injury* […] (P1–Nursing)

*So, I’m going to pass this larger caliber tube* (nasogastric)*; I’m going to use a large caliber vein with the intention that the person learns a lesson and doesn’t try to repeat it next time, as if the pain they were inflicting at that moment was perhaps greater than what they caused themselves.* (P1–Nursing)

In addition to these aspects revealed by the meaning of NSSI, professionals reported difficulty in understanding, communicating with and caring for adolescents, impacting what is perceived as a health need and offered as care:

*It is always a challenge, as I always have difficulty communicating with this group.* […] *these are patients who find it difficult to access information, and most of them are withdrawn.* […] *it is difficult to access health information and history with these companions.* (P4–Nursing)

*The adolescent is very reserved, so he shares very little with us about what makes him do this. He shares very little about the anguish and distress that led to this type of behavior*. (P5–Nursing)

Furthermore, they revealed the need for a broader look at the psychosocial issues of adolescents being treated, highlighting the need for care centered on the person, the family and their life context. These aspects strongly dialogue with the concepts of comprehensive care:

The actions depend a lot on the patient’s profile at the time of the consultation […] t*hey have to be assessed individually so that we can make the best decision, but it is a decision that varies a lot from case to case.* (P6–Medicine)

*We talk a lot about what resentments they feel about this, if they feel angry, if they feel ashamed, how they feel, right?* […] (P7–Medicine)

*Because it involves a adolescent* […] *it is an even more delicate situation because, whenever they arrive, we have to get in touch with their family and, sometimes, it is a fragile family* […] (P8–Nursing)

*We also monitor the family member of the child’s primary caregiver, and we usually call other family members who also live with this patient, right? So, we can investigate the history, investigate what the family structure is like, what the patient’s daily life is like, and what the family thinks about it.* (P5–Nursing)

Participants reported situations in which NSSI can pose a risk to adolescents’ lives and that, in this environment, the focus should be on adolescents, with stabilization of the condition and then understanding the meanings of the act:

*Because if she is in the emergency room, it is because she caused some risk to her life, but we do not know if that risk to her life was intentional, like a real suicide attempt, or perhaps unintentional. She was self-injuring and ended up causing harm to herself that led to a risk to her life, so I think that, perhaps, after stabilizing the patient and talking to him, perhaps, it is really possible to differentiate*. (P1–Nursing)

*The first contact I always have is to show support, not to try to understand right awa*y. (P1–Nursing)

*My actions are to try to provide support, right? Also identify what the immediate triggers of this patient are and, if possible, try to provide long-term support.* (P3–Medicine)

Participants reported the importance of experience and professional preparation, so as to feel confident in providing the humanized and horizontal care that this type of situation demands. They mentioned the need for continuing education and psychological care for professionals working there, considering the comprehensiveness of practices in meetings with adolescents who have self-injured.

*Training of emergency teams is essential; trained teams are needed to treat individuals holistically* […] (P4–Nursing)

*I think that training is always welcome, in the sense of demystifying and beginning to explain what self-injury is* […] (P7–Medicine)

*In the institution where I work, we do not have a psychologist to help us deal with situations like these, neither with the user, the client, nor with us because, many times, as it is a delicate situation, it is also difficult for the healthcare professional.* (P5–Nursing)

2. Beyond that momentary injury – the organization of practices and services for the care of adolescents who have self-injured

This category included reports on how the ES has been organized to provide care for NSSI in adolescence. Firstly, participants reiterated that humanized care and empathy in care are essential. However, according to what can be offered in an emergency environment, which presents characteristics of more punctual and immediate care, this perspective was present in a more in-depth way by nursing professionals:

*Each service must be individualized; following protocols does not help in these cases* […] (P4–Nursing)

*So, I think that holding their hand, touching them, looking them in the eyes, telling them that everything will be okay and showing empathy, telling them that I understand that they are going through a difficult time, but that we will do everything we can to help them, spending quality time with them. So, touching them, holding their hand, looking them in the eye, allowing a moment of silence, perhaps, so that if they want to talk about it at that moment, they feel that they can bring it up or not. And in that first moment, just showing affection and making them feel safe and comfortable with me so that later on, maybe I will feel that I can bring up the subject.* (P1–Nursing)

Among the limitations of the environment, participants listed: reduced time for care, especially pointed out by physicians; lack of a multidisciplinary team to provide support to adolescents; lack of resources, structure and a welcoming place for care, pointed out by nursing, with a managerial view of the service. Such structural and organizational elements, articulated with the challenges of meeting adolescents who have self-injured, emerge as boosters of psychological distress for professionals:

*It’s always a bit distressing, it’s a consultation that isn’t quick and takes a long time* […] (P6–Medicine)

*It requires a conversation, attention that will take a little longer.* (P7–Medicine)

[…] *here we don’t have much structure to deal with these psychiatric conditions, right! Our unit is not physically prepared* […] *it is difficult to contact psychiatry, and more specifically child psychiatry, to help us manage these cases*. (P2–Nursing)

*I believe that, for instance, if we could have a multidisciplinary team, it would be essential.* (P5–Nursing)

As a potential, professionals reported that, in the workplace, there is assistance from specialized professionals who contribute to improving care:

*We always have psychiatric support, which is essential for assessing these patients* […] (P6–Medicine)

*There is a social worker at the ER, so we always contact her in this type of case.* (P8–Nursing)

Another issue addressed was the need to refer adolescents to other services after hospital discharge, in order to ensure the continuous process of monitoring these adolescents with care activities. They pointed out that referral to relevant devices can help adolescents at this time, considering the importance of an aligned and responsible care transition for the comprehensiveness of practices.

*We can refer the patient after stabilization, after discharge to a CAPS* […] (P1–Nursing)

*There is a referral, yes* […] *but always with outpatient monitoring here at the hospital, even with child psychiatry* […] (P2–Nursing)

*And in milder cases, we can also refer the patient to the BHU itself, because some treatments, perhaps antidepressants or milder situations, can also be treated by the BHU clinical physician, but the ideal is for the patient to be followed up by the CAPS, as they have already reached this level of self- injury.* (P1–Nursing)

Such concern with the transition was evident among nursing professionals. Participants brought up the difficulty in carrying out this process, expressing the challenge of providing continuity in care due to the network’s fragility. In most situations, adolescents and families are advised to seek care, without any action of coordination or longitudinality from the service itself:

*I know that here* [work municipality] *adolescents end up being referred to CAPSi; it is a difficult referra*l […] (P7–Medicine)

*Regarding referrals from emergency to CAPS, I have never seen anything like this. I have never seen physicians contact or nurses contact the nearest CAPS to refer the patient to the nearest CAPS. There may have been verbal guidance, but nothing that has actually been discussed between professionals with past cases and everything else to CAPS or to the BHU itself.* (P1–Nursing)

*The adolescent leaves the emergency department with this reference, and leaves with a referral for psychiatric monitoring at the municipal support service. Since our network is not interconnected, I can’t say what the outcome will be after this referral to the CAPS, right? Sometimes, there is a possibility that this adolescent may return to us, but he or she will possibly return in a new, specific situation of self-injury or suicide.* (P8–Nursing)

## DISCUSSION

Care for adolescents with NSSI was revealed by practices permeated by prejudice and stigmas both regarding NSSI and regarding the concept of adolescence. Such aspects challenged the search for comprehensive care, which was situated beyond clinical stabilization towards psychosocial elements. Reckless actions and difficulties in communication, especially due to the particularity of “being an adolescent”, made management more complex, increased by the ES’s hostile environment. Continuing education of professionals regarding these issues became necessary. There is a fragile network articulation, so necessary for care transition to guarantee comprehensiveness, despite the organization proposed by RAUE and RAPS.

In general, nursing and medical professionals revealed similar perspectives when looking at the phenomenon. However, some particularities were identified. The organization of care, service and care transition was deepened by nursing. Medicine, on the other hand, placed greater emphasis on the challenges of initial assessment and duration of care. In this regard, there are singularities in training and action in the face of NSSI that direct these perspectives. Reception is a practice recommended by the Brazilian National Humanization Policy, recognizing what the other brings as a legitimate and unique health need to ensure timely access to care technologies^(16)^. The assessment of adolescents with NSSI requires investigation into their history, previous experiences, risk factors and protective factors, as well as the intentionality, lethality and severity of the harm or injury. Both practices, reception and assessment, must be carried out by all higher education healthcare professionals, with care followed by more specific monitoring for different professional categories^(2,3,4)^. Although nursing has training that covers topics such as service and practice management in greater depth, the relevance of networked care, organized by the entire interprofessional team, will be seen later.

The data from this study revealed that there are still preconceived ideas and stigmatization of these adolescents, which can trigger reckless and negligent attitudes by healthcare professionals. Participants also reported difficulties in addressing NSSI, in addition to the challenges related to caring for the adolescent population. In this perspective, a Brazilian study conducted in the countryside of the state of São Paulo with healthcare and education professionals investigated their conceptions regarding NSSI. It brought results that supported the findings of this study: some professionals demonstrated discomfort regarding the topic as well as statements of trivialization and delegitimization of these behaviors. They also highlighted NSSI as an attempt to draw attention, articulated with the adolescent process, disregarding the legitimacy of these phenomena^(17)^.

Still, when it comes to care, participants draw attention to the importance of a broader view of the psychosocial issues of these adolescents and of care focused on each patient’s specific needs, as well as their family, carried out in a humanized manner. Unhealthy and unsupportive family ties are related to suicidal behavior in adolescents. Although separation from the family nucleus is a natural stage in the development process, it is essential that these young people have the anchoring, support and acceptance in the family context to deal with adverse situations and psychological distress. Professionals involved in the adolescent protection network should guide and support family members to recognize their strategic role in mental healthcare. Furthermore, it is essential to disseminate information that favors adolescents’ comprehensive understanding, reinforcing that their pain should be validated and never minimized^(18)^. It is known that such aspects are relevant in any age group, but due to the peculiar developmental state of adolescents, they become essential.

However, there are challenges faced by emergency sectors in achieving this individualized and humanized care for adolescents and their families. According to the data presented in the results section, only half of interviewees had access to discussions about NSSI in their training. Professionals also mentioned the need for professional training to better address this demand. A Brazilian study supported this deficiency, showing that only one professional had had contact with NSSI in his undergraduate course and no professional had participated in training during his time in service^(17)^. Another study also portrayed the lack of formal training in health courses to provide care to adolescents who enter services due to NSSI situations^(19)^. Here, the authors highlight the importance of building teaching and learning strategies that contribute to the training of healthcare professionals, and also including this topic in the curricula of undergraduate courses.

There is a clear need for initiatives aimed at improving the training of professionals and the preparation of health teams working in ESs with regard to mental health as well as the continuing training of these teams. It is essential to promote the development of scientific knowledge and raise awareness among professionals who deal directly with this demand so that they understand that mental healthcare goes beyond the limits of technical procedures, such as pain relief medication administration. It is important to highlight that these services are part of RAPS, whose main function is stabilization through initial care. However, these services often do not adequately cover the psychosocial dimensions, which can be attributed to the gap in the academic training of professionals regarding mental health specificities^(20)^.

The training perspective of professionals can impact the difficulties in managing the phenomenon and population of this study. There is a lack of knowledge, skills and attitudes appropriate for acting on this topic. This gap is aggravated by the professionals’ training path, often structured under a technical perspective, with a predominant emphasis on individuals’ physiological changes, without broadly considering aspects related to the comprehensiveness of the human being. In this context, continuing education is an essential resource to improve the training of teams in caring for adolescents with mental distress^(21)^, including those with NSSI.

Other challenges inherent to the emergency environment were also found in participants’ statements. Among them are the reduced service time, shortage of a multidisciplinary team to provide support to adolescents, lack of appropriate resources and structure for care, and absence of a welcoming place for care. In this context, a study conducted with healthcare professionals working in emergency units supported these findings, by showing that the shortage of human resources, equipment, consumables, and limited physical infrastructure constitute significant barriers to implementing health actions^(22)^.

This structural and organizational deficiency compromises the quality of care provided, directly interfering in the relationships established between workers and users. Even though hard technologies are present in these environments, they do not replace, nor reduce, the importance of soft and soft-hard technologies in healthcare^(21,23)^. Hard technologies are understood as concrete and finished knowledge structures, such as X-ray machines in emergency settings. Soft-hard technologies are structured methodologies and processes, although they foresee social and human involvement, such as management protocols. Soft technologies involve social relations and the subjectivity embedded in them^(21,23)^. In this context, humanized care, already mentioned, and the organization of processes and protocols need to be considered.

In ESs, professionals perform their duties quickly in decision-making, guided by the need to immediately resolve problems^(22)^. Furthermore, they work in environments that are often physically inadequate, marked by a lack of resources and an intense pace of care, which exposes them to significant physical and psychological demands. In this setting, it is essential to recognize the importance of individual and collective capacity to face and overcome the challenges arising from the various adverse situations experienced daily in these work spaces. Equally important is the development of an institutional stance of attention and care aimed at healthcare professionals themselves, to be promoted by institutions and service leaders^(22)^.

The reports revealed a work environment marked by adverse conditions, characterized by an overload of tasks, the requirement to complete activities in a short time, an insufficient number of professionals on teams, unclear responsibilities, lack of institutional support, inadequate physical space, lack of equipment, among other factors. Such inadequate conditions tend to cause distress among workers and compromise the quality of care provided, a reality that can negatively impact the provision of truly humanized care^(23,24)^.

A recent literature review, based on qualitative data, recommended as a nursing care strategy in ESs the enhancement of the ambiance in waiting rooms, with the use of sensory kits, headphones and modeling clay aimed at welcoming adolescents and young people^(23)^. These aspects are in line with the concept of adolescent-friendly services and practices, which relate to the needs for information, health literacy, accessibility, equity and safety in care settings. Building relationships of trust and comfort are also considered essential for adolescents^(25)^. These elements are linked to the Accelerated Global Action for Adolescent Health, which highlights the importance of prioritizing this population in investments and participation in health^(26)^.

Another aspect related to the one discussed above and brought up by the professionals in this study is that which concerns working healthcare professionals’ mental healthcare. It is known that the health team working in ESs may have their quality of life impaired, since they deal daily with work overload in an exhausting workload, with a shortage of human resources and a lack of professional recognition. In addition, ESs are considered stressful environments, with characteristics such as unpredictability and a high flow of care, demanding organizational skills and team decision-making^(23,27,28)^. Other characteristics of these units that can increase work stress are noise, lack of materials, reduced staff numbers, double work shifts and low pay^(23,27,28)^.

In addition to the issues and difficulties already faced by nurses in ESs, the issue of self-injury and suicide can arouse feelings of concern, helplessness, anxiety, anguish, sadness, frustration, indignation, loss, and fear in professionals^(29)^. Here, we will consider the great difficulty in differentiating between NSSI and attempted suicide as soon as patients arrive at the service, as pointed out by the research participants themselves, since it is necessary, first, to stabilize patients in order to then carry out the work of investigation, intervention and prevention of new situations. Thus, a Korean study^(30)^ analyzed the emotional exhaustion of nursing professionals who work with suicide prevention in mental healthcare services. According to the authors, this exhaustion was related to several factors. Among them were the lack of confidence in their abilities and the perception of their limits in the ability to respond to crisis situations. Regarding the latter, they reported dissatisfaction due to the feeling of always doing the minimum and not having the time or structure to deal with crisis situations due to the great workload. Factors also appeared such as: feeling helpless and frustrated for not being able to help patients; having strong empathy and excessive identification with patients, sharing feelings of sadness and despair with the person being treated; difficulty in understanding patients, opposing suicide in their personal values; and believe that suicidal thoughts of people who make attempts do not improve^(30)^.

It is clear that there is psychological and emotional distress among professionals who work in the emergency sectors and who have, among their demands, those related to mental health, NSSI and suicide attempts, especially when it comes to adolescents. Thus, some authors propose possibilities of mental healthcare for these workers. Some of the initiatives are workplace gymnastics and monitoring of professionals’ mental health, encouraging individual strategies such as leisure with physical exercise, music, therapy, walks, in addition to psychological monitoring^(23,27,28)^. It is also necessary to improve remuneration and value the professionals^(27)^. Other recommendations to improve care for NSSI in adolescence are: structuring the work environment so that professionals have enough time to carry out interventions; promoting educational programs that improve their intervention skills; promoting educational programs that help them maintain a therapeutic distance from patients and regulate and manage their own emotions, avoiding excessive involvement in desperate situations and feelings of sadness; understanding patients more from a psychological than a moral point of view; and helping them deal with their own emotions^(30)^.

Finally, this study revealed the weaknesses of networked action, considering the comprehensiveness of practices. In this way, it is possible, for instance, to carry out coordination and matrix work to identify which cases require individual and specialized attention, and which can benefit from educational and multidisciplinary work and other mental healthcare initiatives. To this end, it is possible to rely on the devices found in RAPS^(31)^.

RAPS have basic, specialized, hospital, emergency and other temporary care services, deinstitutionalization strategies and psychosocial rehabilitation^(11)^. The emergency unit, therefore, has a network of services that can be coordinated and activated for mental healthcare, including in situations of NSSI. Thus, it is important to coordinate work with the different services available in the network of each municipality, in order to develop coordinated care and actions^(32)^, based, for instance, on effective communication between services and the development of Singular Therapeutic Plans for RAPS users^(31)^. In this context, producing adolescent-friendly practices and services in other care areas is essential.

The literature presents some initiatives that make these articulations between institutions possible for the mental healthcare of network users. Among them are joint case studies, systematic intersectoral meetings, shared services and matrix support actions. Intersectoral articulation, with the participation of institutions from other sectors, such as social assistance and education, are indicated as necessary articulations for comprehensive care^(32)^. This provides a global vision and actions directed at the individualities and singularities that demand mental healthcare from RAPS users.

As limitations, the study only investigated nursing and medicine professionals. Today, it is possible to find several healthcare professionals actively working in emergency sectors, either as part of the permanent team of the environment or as a support team through interconsultations. Thus, other professional categories, such as social workers, psychologists, and nutritionists, could be among the professionals interviewed, ensuring an even broader view of the situations and patients. Furthermore, it would be interesting to have the perspective of technical and mid-level professionals, such as those in nursing and reception.

Despite its limitations, this study advances mental health for adolescents with NSSI by identifying the challenges present in a service that has been little studied and articulated with this population. These challenges can be converted into key areas for interventions, such as environmental care and reception/assessment of the situations experienced. It also identifies the importance of thinking about flows and protocols that strengthen and subsidize care transition for adolescents from the ES. Thus, it is necessary for future studies to deepen the potential contributions to nursing in the role of management and articulation of practices and services for adolescent health.

## CONCLUSION

The concept of comprehensiveness allowed us to understand the perspective of both healthcare aimed at adolescents with NSSI in ESs and the organization of services. There are important challenges related to the way of working in these settings and training weaknesses in the topics of mental health and adolescence. Networking is made difficult by this care model, which is often based on the logic of referral.

New ways of providing healthcare to adolescents are needed, geared toward current care demands. This aspect involves everything from training, through active and participatory strategies based on social and epidemiological contexts, to management of care and services based on comprehensiveness. New studies, especially in the Global South, focused on listening to other professionals and adolescents themselves, are recommended.

## Data Availability

The underlying contents of the research are fully incorporated into the manuscript.
