# Supplementary material for: Adolescents with self-injurious behavior in emergency services: a look at comprehensive care
Source: Rev Esc Enferm USP. 2025 Aug 11;59:e20250030. doi: 10.1590/1980-220X-REEUSP-2025-0030en (PMC12345807; doi:10.1590/1980-220X-REEUSP-2025-0030en)
Supplement: Translation

# Translation: Adolescentes com comportamentos autolesivos em serviços de urgência e emergência: um olhar pela integralidade do Cuidado

**DOI:** 10.1590/1980-220X-REEUSP-2025-0030pt

**Authors:** Janiely Aparecida Senne de Sousa Leite, Ana Paula Miranda de Araújo Soares, Nathalia Vitória de Carvalho Martinez, Aline Conceição Silva, Angelina Lettiere-Viana, Tauani Zampieri Fermino, Diene Monique Carlos

**Affiliations:** 1 Universidade Federal de São Carlos, Departamento de Enfermagem, São Carlos, SP, Brasil.; 2 Universidade de São Paulo, Escola de Enfermagem de Ribeirão Preto, Ribeirão Preto, SP, Brasil.; 3 Universidade de São Paulo, Escola de Enfermagem, São Paulo, SP, Brasil.

**Keywords:** Adolescente, Comportamento Autodestrutivo, Enfermagem, Emergências, Pesquisa Qualitativa

## Abstract

**Objetivo:**

Compreender o cuidado com adolescentes com história de autolesão sob a ótica dos profissionais de saúde atuantes em serviços de urgência e emergência.

**Método:**

Pesquisa qualitativa, ancorada no conceito de integralidade do cuidado. Participaram oito profissionais de saúde atuantes em serviços de urgência e emergência, recrutados pela técnica bola de neve. A coleta de dados ocorreu entre 2021 e 2022, a partir de entrevistas semiestruturadas em plataformas virtuais, e os dados foram analisados pela análise temática reflexiva.

**Resultados:**

Emergiram dois temas finais: 1. O encontro com o adolescente que se autolesionou sentimentos relacionados; e 2. Além daquela lesão momentânea a organização das práticas e dos serviços para o cuidado com o adolescente que se autolesionou.

**Conclusão:**

Há desafios relacionados ao modo de trabalho nesses cenários e fragilidades formativas nos temas saúde mental e adolescências. A articulação em rede é dificultada, sendo o cuidado pautado na lógica do encaminhamento. O conceito de integralidade pode ser disparador da organização das ações, dos serviços e das políticas direcionadas a esse fenômeno.

## INTRODUÇÃO

As adolescências vêm se constituindo populações vulneráveis a violências autoinfligidas, com impactos nos perfis de morbimortalidade dessa população^(1)^. As autolesões não suicidas (ALNS) têm se destacado como fenômeno experienciado. Elas são conceituadas como lesões deliberadas que alteram ou destroem o tecido corporal, não sendo socialmente aceitas. A princípio, não há intenção consciente de suicídio, mas podem ser fatores de risco para esse agravo^(2)^.

Estatísticas demonstraram taxas de prevalência de 11,5% a 33,8% de ALNS em países do Sul Global^(3)^. No Brasil, apesar das limitações dos dados estatísticos relacionados a esse fenômeno, há maior incidência de comportamentos autolesivos nos últimos anos^(4)^. O principal método utilizado é o corte nos braços e em outras partes do corpo, podendo também aparecer enquanto queimaduras, principalmente em adolescentes do sexo masculino. Pode se relacionar a sofrimentos psíquicos como depressão, ansiedade e fobia social^(2,3,4,5)^.

Entre os principais riscos desse comportamento em adolescentes, está o de regular as emoções, como raiva, angústia e medo. O singular processo de neurodesenvolvimento na adolescência traz maior responsividade emocional com menor controle e percepção de riscos. Outros fatores de vulnerabilidade identificados na literatura são pertencer ao sexo feminino, sofrer violência, conflitos em relacionamentos afetivo-sexuais e familiares, contato com pares que também se autolesionam, manifestar baixa autoestima, dificuldades de expressar emoções, autocrítica, e irregularidades no sono^(2,5)^.

A literatura vem sinalizando a importância de um manejo clínico da ALNS apropriado, em especial no primeiro acesso, como em serviços de urgência e emergência (UE). Tal aspecto é crucial para reduzir a morbimortalidade da população adolescente e jovem. A despeito disso, o cuidado com a ALNS e disponibilidade de recursos se mantêm planejados e articulados de forma frágil, possivelmente devido à não disposição de estratégias preventivas ou recursos para assinalar elementos estruturais relacionados à ALNS nas adolescências^(3)^. Jovens relatam a necessidade de serviços sensíveis e cuidado compassivo que os vejam como pessoas para além de seus diagnósticos, fazendo-os se sentir seguros, acolhidos e apoiados^(6)^.

Apesar da constante e crescente presença de cuidados voltados a adolescentes em situação de ALNS em serviços de UE, a produção científica é lacunar, em especial em países do Sul Global, sinalizando desafios presentes nesse cenário^(7,8)^. Estudo realizado com enfermeiras na Dinamarca trouxe a percepção do manejo da ALNS como um trabalho desgastante, infinito e frustrante, em especial nos aspectos psicossociais^(7)^. Outro estudo australiano demonstrou a necessidade de educação permanente voltada à construção de relações terapêuticas nesse cuidado^(8)^.

Há um diálogo entre as necessidades de saúde vividas por adolescentes em situação de ALNS e o conceito de integralidade. Como um dos princípios fundamentais previstos no Sistema Único de Saúde (SUS) brasileiro, volta-se à apreensão ampliada das necessidades e consequente adequação de ofertas contextualmente articuladas ao encontro entre a pessoa que busca o serviço e a equipe de saúde. Possui sentidos relacionados às características de políticas que se voltam à articulação de ações preventivas e assistenciais, e às práticas de saúde que se fazem em ato nos serviços e encontros^(9)^. Busca garantir o acesso e a continuidade da assistência nos diferentes pontos das Redes de Atenção à Saúde (RAS) por meio da transição do cuidado. As RAS são modelos organizativos para o SUS, visando articular e integrar os serviços de saúde, incluindo a Rede de Atenção às Urgências e Emergências (RAUE)^(10)^ e a Rede de Atenção Psicossocial (RAPS)^(11)^, que se relacionam ao cuidado com as ALNS.

Diante do exposto, o objetivo deste estudo foi compreender o cuidado com adolescentes com história de autolesão sob a ótica de profissionais de saúde atuantes em serviços de UE. Entende-se que este objeto de estudo, alinhado à perspectiva da enfermagem como prática social, pode permitir avanços na construção de conhecimento científico, prático e gerencial em um campo ainda timidamente ocupado pela agenda da área.

## MÉTODO

### Tipo de Estudo

Pesquisa de abordagem qualitativa^(12)^, ancorada no conceito de integralidade do cuidado^(9)^.

### População e Critérios de Seleção

Foi utilizada a execução da amostragem em bola de neve, que se construiu de maneira a localizar algumas pessoas com o perfil necessário para a pesquisa, por meio de informantes- chave, nomeados como sementes. Assim, as sementes ajudaram o pesquisador a iniciar seus contatos e a tatear o grupo a ser pesquisado. Em seguida, solicitou-se que as pessoas indicadas pelas sementes indicassem novos contatos com as características desejadas, a partir de sua própria rede pessoal e assim por diante, de forma que o quadro de amostragem pudesse crescer a cada entrevista^(13)^. A primeira semente foi uma pessoa membro do grupo de pesquisa da autora principal.

Foram incluídos os profissionais da área da saúde, com educação superior, que já tinham atuado em atendimento de adolescentes com história de autolesão, sobretudo com experiências e vivências em unidades de UE em qualquer esfera dos níveis de atenção à saúde do SUS ou em instituições de saúde suplementar (privada), e que trabalharam nos serviços por, pelo menos, seis meses. Foram excluídos os profissionais que não estivessem em exercício ativo nos serviços durante o período de coleta de dados e não resposta após três tentativas de contato.

Ao longo do processo, 23 pessoas foram contatadas para participar, sendo que cinco recusaram pelo tema do estudo; sete não responderam às tentativas de contato; e três recusaram devido à impossibilidade de tempo. Dessa maneira, oito participantes compuseram este estudo.

### Coleta de Dados

A entrevista semiestruturada^(12)^ foi utilizada como técnica para coleta de dados. Os participantes foram caracterizados mediante questionário sociodemográfico. As entrevistas foram realizadas na modalidade remota, devido à pandemia de COVID-19, e o roteiro abordou percepções sobre as práticas, sentimentos e desafios no cuidado com a ALNS junto a adolescentes. Nesse sentido, foram dadas opções mediadas por plataformas virtuais gratuitas à medida que os participantes se sentissem mais confortáveis. O Termo de Consentimento Livre e Esclarecido foi enviado através do *Google Forms^®^*. As entrevistas foram realizadas por meio de ligação de vídeo pelo aplicativo *WhatsApp^®^*, e duraram em média 25 minutos. Os participantes foram identificados com a letra P, sendo numerados na sequência em que as entrevistas foram realizadas.

O período de coleta se deu entre dezembro de 2021 e julho de 2022. Optou-se neste estudo por buscar a saturação de significado (*meaning saturation*), que corresponde a uma discussão mais profunda, rica em detalhes e complexa com os dados, para assegurar a compreensão de um fenômeno de interesse^(14)^.

### ANÁLISE DE DADOS

Os dados foram analisados mediante a análise temática reflexiva^(15)^. Trata-se de análise com lógica indutiva, a partir dos dados, considerada um processo reflexivo, criativo, subjetivo e deliberativo. Traz uma sistemática e rigorosa aproximação para codificação e criação de temas que é ao mesmo tempo fluida e recursiva. Foram seguidas as seguintes etapas: (I) familiarização com os dados; (II) codificação; (III) busca por temas; (IV) revisão de tema; (V) definição e nomeação dos temas; (VI) escrita final.

### Aspectos Éticos

O estudo seguiu as recomendações da Resolução nº 466/2012 sobre pesquisa envolvendo seres humanos. Foi, inicialmente, aprovado pelos dirigentes municipais e locais dos serviços, sendo aprovado pelo Comitê de Ética em Pesquisa com Seres Humanos da Universidade Federal de São Carlos em 22/08/2019, sob Certificado de Apresentação para Apreciação Ética nº 17176219.6.0000.5504.

## RESULTADOS

Participaram oito profissionais de saúde: dois homens e seis mulheres. Seis profissionais possuíam entre 25 e 30 anos, e dois, entre 35 e 39 anos. Quatro eram casados/as e quatro eram solteiros/as. Sete não possuíam filhos e um participante tinha um filho de 4 anos de idade. Cinco se declararam de cor branca, uma, amarela, e duas, parda. Dois não pertenciam a nenhuma religião; um era espírita; e os demais eram católicos. A formação profissional era composta por cinco enfermeiras, sendo uma com especialização e mestrado, e três médicos (residência em otorrinolaringologia, psiquiatria e pediatria). O tempo de atuação em serviço foi entre seis meses e 11 anos.

Quatro profissionais tiveram contato com o tema da ALNS na sua formação profissional. Três participaram da atualização sobre a temática durante a atuação profissional. Sete profissionais referiram que o tema da adolescência foi abordado durante a graduação.

Os dados advindos das entrevistas semiestruturadas serão apresentados nos temas finais a seguir.

1. O encontro com o adolescente que se autolesionou – sentimentos relacionados

Esta categoria permeou a compreensão dos profissionais diante do encontro com adolescentes nos serviços de UE. Os profissionais trouxeram que existe certa banalização ao cuidado com a ALNS em adolescentes, expondo ideias pré-concebidas sobre a situação, caracterizando-a como busca deliberada por atenção e entendimento de ocuparem espaço de outras pessoas:

[...] *as pessoas têm um pouco de preconceito, a maioria delas. Achando que “poxa” a pessoa está tentando chamar atenção de algum jeito, principalmente quando é uma lesão que não causa risco à vida, então a pessoa tá tentando chamar atenção.* (P1–Enfermagem)

*Infelizmente, muitos profissionais que atendem esses casos vêm como o adolescente, o paciente querendo chamar atenção, enfim.* (P2–Enfermagem)

Um participante trouxe falas sobre a dificuldade de os profissionais entrarem em contato com a ALNS e, a partir disso, buscar compreender a vivência dos adolescentes, pensando a integralidade do cuidado. Compreendeu-se a necessidade do desenvolvimento de habilidades atitudinais que permitam entrar em contato com a dor do outro sem desconsiderar o impacto desses fenômenos à saúde mental dos profissionais:

*É difícil entender o que os adolescentes estão vivendo ou passand*o. […] *é desafiador também porque, dentro da equipe, a gente tem muitas pessoas com muitas culturas diferentes e automutilação; ela é algo violento, é algo chocante, é algo que causa um certo pavor nas pessoas* […] *isso acontece como eu já vi. Eu já vi às vezes em equipes menos treinadas para esse tipo de situação de ter um pavor, uma questão de estigma da autolesão.* (P3–Medicina)

As ações, diante de situações de ALNS trazidas pelos participantes, podem ser permeadas por atitudes agressivas e imprudentes, devido à estigmatização presente, como descrevem as falas a seguir:

[…] *algumas pessoas têm um pouco de preconceito e acaba tratando a pessoa de uma maneira mais agressiva, mas arrogante, por saber que a pessoa causou a própria lesão* […] (P1–Enfermagem)

*Então, vou passar essa sonda mais calibrosa* (nasogástrica)*; vou pegar uma veia de alto calibre com a intenção de que a pessoa aprenda alguma lição e não tente repetir isso da próxima vez, como se aquela dor que eles tivessem infringindo naquele momento, talvez, fosse maior ao que levou ela a causar por ela mesma.* (P1–Enfermagem)

Além desses aspectos desvelados pelo sentido das ALNS, os profissionais relataram dificuldade em compreender, comunicar-se e cuidar da pessoa adolescente, trazendo impacto ao percebido como necessidade de saúde e ofertado como cuidado:

*É sempre um desafio, pois sempre tenho dificuldade em relação à comunicação com esse grupo.* […] *são pacientes difíceis de acessar informações, em sua maioria, retraídos.* […] *sendo difícil acessar informações de saúde e histórico com esses acompanhantes.* (P4–Enfermagem)

*O adolescente é muito fechado, então ele divide muito pouco com a gente o que leva ele fazer isso. Ele compartilha muito pouco das angústias e aflições que levaram a desencadear esse tipo de comportamento.* (P5–Enfermagem)

Ademais, revelaram a necessidade de um olhar ampliado para questões psicossociais dos adolescentes atendidos, trazendo a necessidade do cuidado centrado na pessoa, na família e em seu contexto de vida. Esses aspectos dialogam fortemente com as concepções para o cuidado integral:

*As ações dependem muito do perfil do paciente na hora da consulta* […] *eles têm que ser avaliados individualmente para a gente conseguir tomar a melhor decisão, mas é uma decisão que varia muito caso a caso.* (P6–Medicina)

*A gente conversa bastante sobre quais ressentimentos eles sentem em relação a isso, se sentem raiva, se sentem vergonha, como é que eles se sentem, né* […] (P7–Medicina)

*Por se tratar de adolescente* […] *é uma situação mais delicada ainda porque, sempre quando chega, a gente tem que entrar em contato com a família e, às vezes, é uma família fragilizada* […] (P8–Enfermagem)

*Ainda a gente também faz o acompanhamento do familiar do cuidador principal dessa criança, e a gente costuma chamar outros membros da família que convivam também, né? Com esse paciente, pra gente fazer uma investigação do histórico, fazer uma investigação de como é a estrutura dessa família, como é o dia a dia desse paciente e o que a família pensa sobre isso*. (P5–Enfermagem)

Os participantes relataram situações em que as ALNS podem oferecer risco de vida ao adolescente e que, nesse ambiente, o olhar deve se voltar à pessoa adolescente, com estabilização do quadro e depois compreensão dos significados ao ato:

*Porque se ela “tá” na sala de emergência, porque ela causou algum risco para ela de vida, só que a gente não sabe se aquele risco de vida foi intencional como uma tentativa de suicídio de verdade ou, talvez, não intencional. Ela estava se automutilando e acabou causando um dano para ela que levasse a um risco de vida, então acho que, talvez, depois de estabilizar o paciente e conversar com ele, talvez, tem realmente como diferenciar.* (P1–Enfermagem)

*O primeiro contato que eu tenho sempre é demonstrar apoio, não tentar entender de cara.* (P1–Enfermagem)

*As minhas ações é tentar acolher, né? Identificar também quais são os gatilhos imediatos desse paciente e, se possível, tentar a longo prazo.* (P3–Medicina)

Os participantes relataram a importância da experiência e do preparo profissional, de modo a se sentirem confiantes para prestar o cuidado humanizado e horizontal que demanda esse tipo de situação. Referiram a necessidade de educação permanente e cuidado psicológico aos profissionais que ali atuam, pensando a integralidade das práticas no encontro com a pessoa adolescente que se autolesionou.

*O treinamento das equipes de UE é essencial; são necessárias equipes treinadas para tratar os indivíduos de forma holística* […] (P4–Enfermagem)

*Eu acho que capacitações são sempre bem-vindas, no sentido de desmistificar e começar a explicar mesmo o que é autolesão* […] (P7–Medicina)

*Na instituição em que eu trabalho, nós não temos um psicólogo que nos ajude a lidar com situações como essas, nem com o usuário, o cliente, nem tampouco conosco porque, por diversas vezes, por ser uma situação delicada, é difícil também para o profissional de saúde.* (P5–Enfermagem)

2. Além daquela lesão momentânea – a organização das práticas e dos serviços para o cuidado com o adolescente que se autolesionou

Esta categoria contemplou relatos sobre a forma com que o serviço de UE vem se organizando para cuidado com a ALNS na adolescência. Primeiramente, os participantes reiteraram serem fundamentais o cuidado humanizado e a empatia nos atendimentos. Entretanto, de acordo com o que é possível de ser oferecido em ambiente de UE, que apresenta características de um atendimento mais pontual e imediato, este olhar esteve presente de forma mais aprofundada pelos profissionais de enfermagem:

*Cada atendimento deve ser individualizado; seguir protocolos não ajuda nesses casos* […] (P4–Enfermagem)

*Então, eu acho que segurar na mão, tocar nele olhar nos olhos dizer que vai ficar tudo bem e demonstrar essa empatia, falar para ele que eu entendo que ele “tá” passando por um momento difícil, mas que a gente vai fazer o possível para poder ajudar ele, despender um tempo de qualidade com a pessoa. Então, tocar nele, segurar na mão, olhar no olho, permitir um momento de silêncio, talvez, para que se ele quiser falar sobre aquilo naquele momento, ele sente que pode tocar no assunto ou não. E nesse primeiro momento, só demonstrar afeição e fazer com que ele se sinta seguro e à vontade comigo para depois no momento posterior talvez eu sentir que eu possa tocar nesse assunto.* (P1–Enfermagem)

Entre as limitações do ambiente, os participantes listaram: tempo reduzido de atendimento, em especial apontado pelos médicos; escassez da equipe multiprofissional para prestar suporte aos adolescentes; falta de recursos, estrutura e local acolhedor para o atendimento, apontados pela enfermagem, com olhar gerencial ao serviço. Tais elementos estruturais e organizacionais, articulados aos desafios do encontro com o adolescente que se autolesionou, emergem como potencializadores de sofrimento psíquico dos profissionais:

*É sempre meio angustiante, é uma consulta que não é rápida que demora* […] (P6–Medicina)

*Exige uma conversa, uma atenção que vai demandar um tempo um pouquinho maior*. (P7–Medicina)

[…] *aqui a gente não tem muita estrutura para atender esses quadros psiquiátricos, né! A nossa unidade não é fisicamente preparada* […] *tem uma dificuldade de contatar a psiquiatria, e mais especificamente a psiquiatria infantil, para ajudar a gente a conduzir esses casos.* (P2–Enfermagem)

Acredito que, por exemplo, se a gente tivesse como ter uma equipe multidisciplinar, é fundamental. (P5–Enfermagem)

Como potencialidade, os profissionais relataram que, no local de trabalho, existe o auxílio de profissionais especializados que contribuem para melhoria do cuidado:

*A gente sempre tem uma retaguarda de psiquiatria, que é essencial para fazer uma avaliação desses pacientes* […] (P6–Medicina)

*Tem uma assistente social no PA, então a gente sempre aciona ela nesse tipo de caso.* (P8–Enfermagem)

Outra questão abordada foi a necessidade do referenciamento dos adolescentes para outros serviços após a alta hospitalar, no intuito de garantir o processo contínuo de acompanhamento desse adolescente com atividades de cuidado. Eles trouxeram que o encaminhamento para dispositivos pertinentes pode auxiliar os adolescentes nesse momento, pensando a importância de uma transição de cuidado alinhada e responsável para a integralidade das práticas.

*A gente pode encaminhar o paciente depois de estabilizado, depois da alta para um CAPS* […] (P1–Enfermagem)

*Existe o encaminhamento sim* […] *mas sempre com acompanhamento ambulatorial aqui no hospital mesmo com a psiquiatria infantil* […] (P2–Enfermagem)

*E em casos mais leves, também, a gente pode encaminhar até para própria UBS, porque alguns tratamentos, talvez antidepressivos ou de situações mais leves, podem ser tratados também pelo médico clínico da UBS, mas o ideal mesmo é que ele seja acompanhado pelo CAPS, por já terem chegado nesse nível de autoagressão*. (P1–Enfermagem)

Vislumbrou-se tal preocupação com a transição pelos profissionais de enfermagem. Os participantes trouxeram a dificuldade em realizar este processo, manifestando o desafio em dar continuidade no cuidado devido à fragilidade da rede. Na maior parte das situações, o adolescente e a família são orientados a buscar cuidado, sem nenhuma ação de articulação ou longitudinalidade do próprio serviço:

*Eu sei que aqui* [município de trabalho] *os adolescentes acabam sendo encaminhados para o CAPSi; é um encaminhamento difícil* […] (P7–Medicina)

*Com relação ao encaminhamento da UE para CAPS, eu nunca presenciei. Nunca vi médicos entrar em contato, enfermeira entrar em contato com CAPS mais próximo para encaminhar o paciente passar o caso. Talvez tem tido orientações verbais, mas nada que realmente tem sido conversado entre profissionais com caso passado e tudo mais para CAPS ou para própria UBS.* (P1–Enfermagem)

*O adolescente, ele sai no serviço de urgência e emergência com essa referência, ele sai com encaminhamento para acompanhamento psiquiátrico no serviço de suporte do município. Como nossa rede não é interligada, eu não sei dizer posterior esse encaminhamento ao CAPS qual o desfecho disso, né? Às vezes, existem a possibilidade de que esse adolescente possa retornar para nós, mas ela retornará possivelmente numa nova situação pontual de automutilação ou de suicídio.* (P8–Enfermagem)

## DISCUSSÃO

O cuidado com o adolescente em situação de ALNS foi desvelado por práticas permeadas por preconceitos e estigmas tanto diante da ALNS quanto diante da concepção de adolescência. Tais aspectos desafiaram a busca pela integralidade no cuidado, que foi situada para além da estabilização clínica para elementos psicossociais. Ações imprudentes e dificuldades na comunicação, especialmente pela particularidade de “ser adolescente”, tornaram o manejo mais complexo, acrescido pelo ambiente hostil da UE. A Educação Permanente dos profissionais diante desses temas se fez necessária. Há uma articulação em rede frágil, tão necessária à transição do cuidado para garantir a integralidade, a despeito da organização proposta pela RAUE e RAPS.

No geral, os profissionais de enfermagem e medicina desvelaram perspectivas semelhantes no olhar ao fenômeno. Entretanto, delimitaram-se algumas particularidades. A organização da assistência, do serviço e da transição de cuidado foi aprofundada pela enfermagem. Já a medicina denotou maior ênfase aos desafios da avaliação inicial e duração dos atendimentos. Neste sentido, há singularidades formativas e de atuação diante da ALNS que direcionam tais perspectivas. O acolhimento é uma prática recomendada pela Política Nacional de Humanização, reconhecendo o que o outro traz como legítima e singular necessidade de saúde para garantir o acesso oportuno às tecnologias de cuidado^(16)^. A avaliação do adolescente em situação de ALNS demanda investigação sobre o histórico, experiências prévias, fatores de risco e fatores de proteção, bem como a intencionalidade, a letalidade e a gravidado do dano ou lesão. Ambas as práticas, acolhimento e avaliação, devem ser realizadas por todos os profissionais de saúde de nível superior, sendo os cuidados seguidos de acompanhamento mais específico a diferentes categorias profissionais^(2–4)^. Apesar de a enfermagem possuir formação que contempla mais profundamente temas como gestão de serviços e práticas, será vista adiante a relevância do cuidado em rede, organizado por toda equipe interprofissional.

Os dados deste estudo revelaram que ainda há ideias pré- concebidas e de estigmatização desses adolescentes, o que pode desencadear atitudes imprudentes e negligentes por profissionais de saúde. Os participantes relataram, também, dificuldades de abordar o tema ALNS, além dos desafios relativos ao cuidado com a população adolescente. Nessa perspectiva, estudo brasileiro realizado no interior do estado de São Paulo com profissionais de saúde e de educação investigou suas concepções a respeito da ALNS. Trouxe resultados que corroboraram os achados no presente estudo: alguns profissionais demonstraram desconforto em relação ao tema, assim como falas de banalização e deslegitimação desses comportamentos. Destacaram, também, a ALNS como tentativa de chamar atenção, articulada ao processo adolescente, desconsiderando a legitimidade desses fenômenos^(17)^.

Ainda assim, quando se trata do cuidado, os participantes chamam a atenção para a importância de um olhar ampliado para as questões psicossociais desses adolescentes e de cuidado centrado nas necessidades específicas de cada paciente, assim como de sua família, realizado de forma humanizada. Vínculos familiares pouco saudáveis e sustentadores se relacionam a comportamentos suicidas adolescentes. Embora o afastamento do núcleo familiar seja uma etapa natural do processo de desenvolvimento, é fundamental que esses jovens tenham a ancoragem, o suporte e o acolhimento no contexto da família para lidar com situações adversas e sofrimento psíquico. Profissionais inseridos na rede de proteção à adolescência devem orientar e apoiar os familiares a reconhecer seu papel estratégico no cuidado com a saúde mental. Além disso, é imprescindível que haja disseminação de informações que favoreçam a compreensão integral do adolescente, reforçando que sua dor deve ser validada e jamais minimizada^(18)^. Sabe-se que tais aspectos são relevantes em quaisquer faixas etárias, mas pelo peculiar estado desenvolvimental de adolescentes, tornam-se primordiais.

No entanto, há desafios encontrados nos setores de UE para alcançar esse cuidado individualizado e humanizado ao adolescente e seus familiares. Segundo os dados expostos na seção de resultados, apenas metade dos entrevistados teve acesso a discussões sobre ALNS em suas formações. Os profissionais trazem, ainda, a necessidade de capacitação dos profissionais para melhor atuação em relação a essa demanda. Estudo brasileiro corroborou tal deficiência, mostrando que apenas um profissional havia tido contato com o tema da ALNS em seu curso de graduação e nenhum profissional havia participado de formações durante o seu tempo de serviço^(17)^. Outro estudo também retratou a ausência de formação formal em cursos de saúde para prestar cuidados a adolescentes que dão entrada nos serviços devido às situações de ALNS^(19)^. Aqui, os autores destacam a importância de construir estratégias de ensino e aprendizagem que contribuam para a formação dos profissionais de saúde, e também incluir esse tema nas grades curriculares dos cursos de graduação.

Evidencia-se a necessidade de iniciativas voltadas à melhoria da formação dos profissionais e do preparo das equipes de saúde que atuam em serviços de UE no que se refere à saúde mental, bem como à capacitação contínua dessas equipes. É fundamental promover o desenvolvimento do conhecimento científico e sensibilizar os profissionais que lidam diretamente com essa demanda, para que compreendam que o atendimento em saúde mental ultrapassa os limites dos procedimentos técnicos, como a administração de medicamentos para alívio da dor. É importante destacar que esses serviços integram a RAPS, cuja função principal é a estabilização por meio de cuidados iniciais. No entanto, esses serviços frequentemente não contemplam adequadamente as dimensões psicossociais, o que pode ser atribuído à lacuna existente na formação acadêmica dos profissionais em relação às especificidades da saúde mental^(20)^.

A perspectiva formativa dos profissionais pode impactar as dificuldades no manejo do fenômeno e população deste estudo. Observa-se uma carência de conhecimentos, habilidades e atitudes adequadas para a atuação diante dessa temática. Tal lacuna é agravada pelo próprio percurso formativo dos profissionais, frequentemente estruturado sob uma perspectiva tecnicista, com ênfase predominante nas alterações fisiológicas dos indivíduos, sem considerar, de forma ampla, os aspectos relacionados à integralidade do ser humano. Nesse contexto, a educação permanente configura-se um recurso essencial para aprimorar a capacitação das equipes no cuidado com adolescentes em sofrimento psíquico^(21)^, incluindo aqueles em situação de ALNS.

Outros desafios inerentes ao ambiente de UE também foram encontrados entre as falas dos participantes. Entre eles, estão o tempo reduzido de atendimento, escassez da equipe multiprofissional para prestar suporte aos adolescentes, falta de recursos e estrutura apropriados para o atendimento, e ausência de um local acolhedor para o atendimento. Nesse contexto, estudo conduzido com profissionais de saúde atuantes em unidades de UE corroborou tais achados, ao evidenciar que a escassez de recursos humanos, equipamentos, materiais de consumo e a limitação da infraestrutura física se configuram barreiras significativas à efetivação das ações de saúde^(22)^.

Essa carência estrutural e organizacional compromete a qualidade dos atendimentos prestados, interferindo diretamente nas relações estabelecidas entre trabalhadores e usuários. Ainda que haja a presença de tecnologias duras nesses ambientes, elas não substituem, tampouco reduzem, a importância das tecnologias leves e leve-duras no cuidado em saúde^(21,23)^. Tecnologias duras são entendidas como estruturas de conhecimento concretas e acabadas, como os aparelhos de raio-X em cenários de UE. As tecnologias leve-duras são metodologias e processos estruturados, apesar de prever envolvimento social e humano, como protocolos gerenciais. Já as tecnologias leves envolvem as relações sociais e subjetividade incorporada nelas^(21,23)^. Nesse âmbito, o cuidado humanizado, já citado, e a organização de processos e protocolos precisam ser vislumbrados.

Nos serviços de UE, os profissionais exercem suas funções com rapidez na tomada de decisão, orientando-se pela necessidade de resolução imediata dos problemas^(22)^. Ademais, atuam em ambientes frequentemente inadequados do ponto de vista físico, marcados pela escassez de recursos e por um ritmo intenso de atendimentos, o que os expõe a demandas físicas e psíquicas significativas. Nesse cenário, torna-se essencial reconhecer a importância da capacidade individual e coletiva para enfrentar e superar os desafios decorrentes das diversas situações adversas vivenciadas cotidianamente nesses espaços de trabalho. Igualmente relevante é o desenvolvimento de uma postura institucional de atenção e cuidado voltada aos próprios profissionais de saúde, a ser promovida pelas instituições e pelas lideranças dos serviços^(22)^.

Os relatos evidenciaram um ambiente de trabalho marcado por condições adversas, caracterizado pela sobrecarga de tarefas, exigência de cumprimento de atividades em tempo reduzido, número insuficiente de profissionais nas equipes, indefinição de atribuições, ausência de respaldo institucional, inadequações no espaço físico, carência de equipamentos, entre outros fatores. Tais condições inadequadas tendem a provocar sofrimento entre os trabalhadores e comprometem a qualidade do atendimento prestado, realidade que pode interferir negativamente na oferta de uma assistência verdadeiramente humanizada^(23,24)^.

Revisão de literatura recente, com base em dados qualitativos, recomendou como estratégia de cuidado de enfermagem em serviços de UE a valorização da ambiência nas salas de espera, com a utilização de kits sensoriais, fones de ouvido e massas de modelar voltados ao acolhimento de adolescentes e jovens^(23)^. Esses aspectos dialogam com o conceito de serviços e práticas amigáveis a adolescentes, que se relacionam às necessidades de informação, letramento em saúde, acessibilidade, equidade e segurança nos contextos de cuidado. A construção de relações de confiança e conforto também são colocados como essenciais para adolescentes^(25)^. Tais elementos se articulam com a Ação Global Acelerada para a Saúde de Adolescentes, que sinaliza a importância desta população ser priorizada nos investimentos e participação em saúde^(26)^.

Outro aspecto que se relaciona ao discutido anteriormente e que foi trazido pelos profissionais deste estudo é aquele que diz respeito ao cuidado em saúde mental dos profissionais de saúde atuantes. Sabe-se que a equipe de saúde que atua em serviços de UE pode ter sua qualidade de vida prejudicada, uma vez que lida diariamente com sobrecarga de trabalho em uma carga horária exaustiva, com escassez de recursos humanos e falta de reconhecimento profissional. Além disso, as UEs são tidas como ambientes estressantes, com características como imprevisibilidade e alto fluxo de atendimentos, demandando capacidade de organização e tomada de decisão em equipe^(23,27,28)^. Outras características dessas unidades que podem aumentar o estresse do trabalho são o barulho, falta de materiais, redução dos números de profissionais, dupla jornada de trabalho e baixa remuneração^(23,27,28)^.

Além das questões e dificuldades já enfrentadas pelos enfermeiros em UE, a temática da autolesão e suicídio pode suscitar nos profissionais sentimentos de preocupação, impotência, ansiedade, angústia, tristeza, frustração, indignação, perda e receio^(29)^. Aqui, será considerada a grande dificuldade de diferenciar a ALNS e a tentativa de suicídio logo que o paciente chega ao serviço, como pontuado pelos próprios participantes da pesquisa, já que é necessário, em um primeiro momento, estabilizar o paciente para, então, fazer o trabalho de investigação, intervenção e prevenção de novas situações. Nesse sentido, estudo coreano^(30)^ analisou o esgotamento emocional de profissionais de enfermagem que trabalham com prevenção de suicídio em serviços de saúde mental. Segundo os autores, esse esgotamento estava relacionado a alguns fatores. Entre eles, estavam a falta de confiança em suas habilidades e a percepção de seus limites na capacidade de resposta às situações de crise. Em relação ao último, relataram insatisfação pela sensação de estar sempre fazendo o mínimo e não ter tempo ou estrutura para lidar com situações de crise devido à grande sobrecarga de trabalho. Também apareceram fatores como: se sentir impotente e frustrado por não conseguir ajudar o paciente; ter forte empatia e identificação excessiva com o paciente, compartilhando os sentimentos de tristeza e desespero com a pessoa que está sendo atendida; dificuldade de compreensão em relação ao paciente, opondo-se ao suicídio em seus valores pessoais; e acreditar que os pensamentos suicidas de pessoas que fazem tentativas não melhoram^(30)^.

Percebe-se que existe sofrimento psicológico e emocional de profissionais que atuam em setores de UE e que possuem, entre suas demandas, aquelas relacionadas à saúde mental, ALNS e tentativas de suicídio, em especial quando se trata de adolescentes. Assim, alguns autores propõem possibilidades de cuidado em saúde mental para esses trabalhadores. Algumas das iniciativas são a ginástica laboral e o monitoramento da saúde mental dos profissionais, estimulando estratégias individuais como lazer com exercício físico, música, terapia, caminhadas, além de acompanhamento psicológico^(23,27,28)^. É necessário também melhorar a remuneração e valorizar o profissional^(27)^. Outras recomendações para aprimorar o cuidado com a ALNS nas adolescências são: estruturar o ambiente de trabalho para que os profissionais tenham tempo hábil para fazer as intervenções; promover programas educacionais que melhorem suas habilidades de intervenção; promover programas educacionais que os ajude a manter uma distância terapêutica do paciente e a regular e gerenciar suas próprias emoções, evitando um envolvimento excessivo nas situações desesperadoras e no sentimento de tristeza; compreender os pacientes mais do ponto de vista psicológico do que moral; e ajudar a lidar com suas próprias emoções^(30)^.

Por fim, emergiram neste estudo as fragilidades para a atuação em rede, considerando a integralidade das práticas. Dessa forma, é possível, por exemplo, fazer um trabalho de articulação e matriciamento para que se identifiquem quais são os casos que precisam de atenção individual e especializada, e quais podem se beneficiar de trabalhos educativos e multiprofissionais e outras iniciativas de cuidados em saúde mental. Para isso, é possível contar com os dispositivos encontrados nas RAPS^(31)^.

As RAPS contam com serviços de atenção básica, especializada, hospitalar, de UE e outros serviços de acolhimento transitório, estratégias de desinstitucionalização e reabilitação psicossocial^(11)^. A unidade de UE, portanto, conta com uma rede de serviços que podem ser articulados e acionados para o cuidado em saúde mental, inclusive em situações de ALNS. Assim, é importante articular um trabalho em conjunto com os diferentes serviços disponíveis na rede de cada município, a fim de desenvolver cuidados e ações coordenadas^(32)^, a partir, por exemplo, da comunicação efetiva entre os serviços e da elaboração de Planos Terapêuticos Singulares aos usuários da RAPS^(31)^. Neste âmbito, produzir práticas e serviços amigáveis a adolescentes em outros pontos de atenção se faz essencial.

A literatura traz algumas iniciativas que tornam possível essas articulações entre instituições para o cuidado em saúde mental dos usuários da rede. Entre elas, estão estudos de caso em conjunto, reuniões sistemáticas intersetoriais, atendimentos compartilhados e ações de apoio matricial. A articulação intersetorial, com a participação de instituições de outros setores, como a assistência social e educação, são apontadas como articulações necessárias para a integralidade do cuidado^(32)^. Isso propicia uma visão global e ações direcionadas às individualidades e singularidades que demandam os cuidados em saúde mental dos usuários da RAPS.

Como limitações, o estudo traz a investigação apenas com profissionais das áreas da enfermagem e medicina. Hoje, é possível encontrar diversos profissionais de saúde atuando ativamente em setores de UE, seja como parte da equipe fixa do ambiente ou como equipe de apoio a partir de interconsultas. Dessa forma, outras categorias profissionais, como assistentes sociais, psicólogos, nutricionistas, poderiam estar entre os profissionais entrevistados, garantindo um olhar ainda mais amplo a respeito das situações e dos pacientes. Ademais, seria interessante o olhar de profissionais de nível técnico e médio, como da área da enfermagem, e recepção.

A despeito das limitações, este estudo avança na área da saúde mental do adolescente em situação de ALNS, ao identificar os desafios presentes em um serviço pouco estudado e articulado a esta população. Estes desafios podem ser convertidos em áreas-chave para intervenções, como cuidados com a ambiência e acolhimento/avaliação das situações vividas. Ainda identifica a importância de pensar fluxos e protocolos que fortaleçam e subsidiem a transição de cuidado dos adolescentes a partir da UE. Dessa forma, é necessário que estudos futuros possam aprofundar as potenciais contribuições para a área da enfermagem no papel de gestão e articulação de práticas e serviços para a saúde do adolescente.

## CONCLUSÃO

O conceito de integralidade permitiu compreender o olhar tanto ao cuidado em saúde voltado a adolescentes em situação de ALNS em serviços de UE, quanto à organização dos serviços. Há desafios importantes relacionados ao modo de trabalho nesses cenários e fragilidades formativas nos temas saúde mental e adolescências. A articulação em rede é dificultada a partir desse modelo de cuidado, muitas vezes, pautado na lógica do encaminhamento.

São necessárias novas formas de produção de saúde aos adolescentes voltadas às atuais demandas de cuidado. Esse aspecto envolve desde uma formação, por meio de estratégias ativas e participativas a partir de contextos sociais e epidemiológicos, até uma gestão do cuidado e dos serviços pautada de fato na integralidade. Novos estudos, em especial no Sul Global, voltados a ouvir outros profissionais e os próprios adolescentes, são recomendados.

## Data Availability

Os conteúdos subjacentes ao texto da pesquisa estão contidos no manuscrito.

# Translation: Adolescentes con conductas autolesivas en servicios de urgencias: una mirada a la atención integral

**DOI:** 10.1590/1980-220X-REEUSP-2025-0030es

**Authors:** Janiely Aparecida Senne de Sousa Leite, Ana Paula Miranda de Araújo Soares, Nathalia Vitória de Carvalho Martinez, Aline Conceição Silva, Angelina Lettiere-Viana, Tauani Zampieri Fermino, Diene Monique Carlos

**Affiliations:** 1 Universidade Federal de São Carlos, Departamento de Enfermagem, São Carlos, SP, Brasil.; 2 Universidade de São Paulo, Escola de Enfermagem de Ribeirão Preto, Ribeirão Preto, SP, Brasil.; 3 Universidade de São Paulo, Escola de Enfermagem, São Paulo, SP, Brasil.

**Keywords:** Adolescente, Conducta Autodestructiva, Enfermería, Urgencias Médicas, Investigación Cualitativa

## Abstract

**Objetivo:**

Comprender la atención brindada a adolescentes con antecedentes de autolesión desde la perspectiva de profesionales de la salud que trabajan en servicios de urgencias.

**Método:**

Investigación cualitativa, basada en el concepto de atención integral. Participaron ocho profesionales de la salud que trabajan en servicios de urgencias, reclutados mediante la técnica de bola de nieve. La recopilación de datos se realizó entre 2021 y 2022, mediante entrevistas semiestructuradas en plataformas virtuales, y se analizaron mediante análisis temático reflexivo.

**Resultados:**

Surgieron dos temas finales: 1. El encuentro con el adolescente autolesionado: sentimientos relacionados; y 2. Más allá de esa lesión momentánea: la organización de prácticas y servicios para la atención del adolescente autolesionado.

**Conclusión:**

Existen desafíos relacionados con la forma de trabajar en estos escenarios y deficiencias en la formación en salud mental y adolescencia. El trabajo en red es difícil y la atención se basa en la lógica de la derivación. El concepto de integralidad puede impulsar la organización de acciones, servicios y políticas dirigidas a este fenómeno.

## INTRODUCCIÓN

Los adolescentes están convirtiéndose progresivamente en una población vulnerable a la violencia autoinfligida, lo que produce repercusiones en los perfiles de morbilidad y mortalidad de este grupo^(1)^. Las autolesiones no suicidas (ALNS) se han destacado como un fenómeno frecuente. Se definen como lesiones deliberadas que alteran o destruyen el tejido corporal y no son socialmente aceptadas. En principio, no hay intención consciente de suicidio, pero pueden ser factores de riesgo para esta afección^(2)^.

Las estadísticas han demostrado tasas de prevalencia de ALNS del 11,5% al 33,8% en países del Sur Global^(3)^. En Brasil, a pesar de las limitaciones de los datos estadísticos relacionados con este fenómeno, hay una mayor incidencia de comportamientos autolesivos en los últimos años^(4)^. El método principal utilizado es el corte en los brazos y otras partes del cuerpo, pudiendo también aparecer como quemaduras, principalmente en adolescentes varones. Puede estar relacionado con sufrimientos psíquicos como depresión, ansiedad y fobia social^(2,3,4,5)^.

Entre los principales riesgos de este comportamiento en adolescentes se encuentra el de regular las emociones, como la ira, la angustia y el miedo. El singular proceso de neurodesarrollo en la adolescencia conlleva una mayor responsividad emocional con menor control y percepción de los riesgos. Otros factores de vulnerabilidad identificados en la literatura son pertenecer al sexo femenino, sufrir violencia, conflictos en las relaciones afectivas y sexuales y familiares, contacto con compañeros que también se autolesionan, manifestar baja autoestima, dificultades para expresar emociones, autocrítica e irregularidades en el sueño^(2,5)^.

La literatura ha señalado la importancia de un manejo clínico adecuado de la ALNS, especialmente en el primer acceso, como en los servicios de urgencia y emergencia (UE). Este aspecto es crucial para reducir la morbimortalidad de la población adolescente y joven. A pesar de ello, la atención a la ALNS y la disponibilidad de recursos siguen estando planificadas y articuladas de forma frágil, posiblemente debido a la falta de estrategias preventivas o de recursos para señalar elementos estructurales relacionados con la ALNS en la adolescencia^(3)^. Los jóvenes relatan la necesidad de servicios sensibles y cuidados compasivos que los vean como personas más allá de sus diagnósticos, haciéndoles sentir seguros, acogidos y apoyados^(6)^.

A pesar de la presencia constante y creciente de cuidados dirigidos a adolescentes en situación de ALNS en los servicios de urgencias, la producción científica es deficiente, especialmente en los países del Sur Global, lo que pone de manifiesto los retos que se plantean en este escenario^(7,8)^. Un estudio realizado con enfermeras en Dinamarca reveló la percepción de que el manejo de la ALNS es un trabajo agotador, interminable y frustrante, especialmente en los aspectos psicosociales^(7)^. Otro estudio australiano demostró la necesidad de una educación permanente orientada a la construcción de relaciones terapéuticas en esta atención^(8)^.

Existe un diálogo entre las necesidades de salud que experimentan los adolescentes en situación de ALNS y el concepto de integralidad. Como uno de los principios fundamentales previstos en el Sistema Único de Salud (SUS) brasileño, se centra en una comprensión ampliada de las necesidades y la consiguiente adecuación de las ofertas contextualmente articuladas para el encuentro entre la persona que busca el servicio y el equipo de salud. Tiene significados relacionados con las características de las políticas que se centran en la articulación de acciones preventivas y asistenciales, y con las prácticas de salud que se llevan a cabo en los servicios y encuentros^(9)^. Busca garantizar el acceso y la continuidad de la asistencia en los diferentes puntos de las Redes de Atención a la Salud (RAS) mediante la transición de la atención. Las RAS son modelos organizativos del SUS, cuyo objetivo es articular e integrar los servicios de salud, incluyendo la Red de Atención a Urgencias y Emergencias (RAUE)^(10)^ y la Red de Atención Psicosocial (RAPS)^(11)^, que se relacionan con la atención a las ALNS.

Ante lo expuesto, el objetivo de este estudio fue comprender la atención a adolescentes con antecedentes de autolesiones desde la perspectiva de los profesionales de la salud que trabajan en los servicios de urgencias. Se entiende que este objeto de estudio, en línea con la perspectiva de la enfermería como práctica social, puede permitir avances en la construcción de conocimientos científicos, prácticos y gerenciales en un campo aún poco explorado por la agenda del área.

## MÉTODO

### Tipo de Estudio

Investigación de enfoque cualitativo^(12)^, basada en el concepto de integralidad de la atención^(9)^.

### Población y Criterios de Selección

Se utilizó el muestreo en bola de nieve, que se construyó de manera que se localizara a algunas personas con el perfil necesario para la investigación, a través de informantes clave, denominados semillas. Así, las semillas ayudaron al investigador a iniciar sus contactos y a tantear el grupo a investigar. A continuación, se pidió a las personas indicadas por las semillas que indicaran nuevos contactos con las características deseadas, a partir de su propia red personal, y así sucesivamente, de modo que el cuadro de muestreo pudiera crecer con cada entrevista^(13)^. La primera semilla fue una persona miembro del grupo de investigación de la autora principal.

Se incluyeron profesionales del área de la salud, con educación superior, que ya habían trabajado en la atención de adolescentes con antecedentes de autolesión, sobre todo con experiencias y vivencias en unidades de urgencias en cualquier ámbito de los niveles de atención de salud del SUS o en instituciones de salud complementaria (privada), y que habían trabajado en los servicios durante al menos seis meses. Se excluyó a los profesionales que no estaban en ejercicio activo en los servicios durante el período de recolección de datos y que no respondieron tras tres intentos de contacto.

A lo largo del proceso, se contactaron 23 personas para participar, de las cuales cinco rechazaron la propuesta debido al tema del estudio; siete no respondieron a los intentos de contacto; y tres rechazaron la propuesta por falta de tiempo. De este modo, ocho participantes formaron parte de este estudio.

### Recolección de Datos

Se utilizó la entrevista semiestructurada^(12)^ como técnica de recolección de datos. Los participantes fueron caracterizados mediante un cuestionario sociodemográfico. Las entrevistas se realizaron de forma remota, debido a la pandemia de COVID-19, y el guion abordó percepciones sobre las prácticas, los sentimientos y los desafíos en la atención de la ALNS junto a adolescentes. En este sentido, se ofrecieron opciones mediadas por plataformas virtuales gratuitas para que los participantes se sintieran más cómodos. El Término de Consentimiento Libre y Esclarecido se envió a través de *Google Forms^®^*. Las entrevistas se realizaron mediante videollamada a través de la aplicación *WhatsApp^®^* y duraron una media de 25 minutos. Los participantes fueron identificados con la letra P y numerados en el orden en que se realizaron las entrevistas.

El período de recolección se llevó a cabo entre diciembre de 2021 y julio de 2022. En este estudio se optó por buscar la saturación de significado (*meaning saturation*), que corresponde a una discusión más profunda, rica en detalles y compleja con los datos, para asegurar la comprensión de un fenómeno de interés^(14)^.

### Análisis de Datos

Los datos se analizaron mediante el análisis temático reflexivo^(15)^. Se trata de un análisis con lógica inductiva, a partir de los datos, considerado un proceso reflexivo, creativo, subjetivo y deliberativo. Aporta un enfoque sistemático y riguroso para la codificación y la creación de temas que es a la vez fluido y recursivo. Se siguieron las siguientes etapas: (I) familiarización con los datos; (II) codificación; (III) búsqueda de temas; (IV) revisión de temas; (V) definición y denominación de los temas; (VI) redacción final.

### Aspectos Éticos

El estudio siguió las recomendaciones de la Resolución n.º 466/2012 sobre investigación con seres humanos. Inicialmente, fue aprobado por los responsables municipales y locales de los servicios, y posteriormente por el Comité de Ética en Investigación con Seres Humanos de la Universidad Federal de São Carlos el 22/08/2019, bajo el Certificado de Presentación para Apreciación Ética n.º 17176219.6.0000.5504.

## RESULTADOS

Participaron ocho profesionales de la salud: dos hombres y seis mujeres. Seis profesionales tenían entre 25 y 30 años, y dos, entre 35 y 39 años. Cuatro estaban casados y cuatro eran solteros. Siete no tenían hijos y un participante tenía un hijo de 4 años.

Cinco se declararon de raza blanca, uno, amarilla, y dos, mestizos. Dos no pertenecían a ninguna religión; uno era espiritista; y los demás eran católicos. La formación profesional estaba compuesta por cinco enfermeras, una de ellas con especialización y maestría, y tres médicos (residencia en otorrinolaringología, psiquiatría y pediatría). El tiempo de actuación en el servicio fue de entre seis meses y 11 años.

Cuatro profesionales tuvieron contacto con el tema de la ALNS en su formación profesional. Tres participaron en la actualización sobre el tema durante su actuación profesional. Siete profesionales mencionaron que el tema de la adolescencia fue abordado durante la graduación.

Los datos obtenidos de las entrevistas semiestructuradas se presentarán en los temas finales a continuación.

1. El encuentro con el adolescente que se autolesionó: sentimientos relacionados

Esta categoría permeó la comprensión de los profesionales ante el encuentro con adolescentes en los servicios de urgencias. Los profesionales señalaron que existe cierta banalización en la atención a la ALNS en adolescentes, exponiendo ideas preconcebidas sobre la situación, caracterizándola como una búsqueda deliberada de atención y comprensión por ocupar el espacio de otras personas:

[...] *la mayoría de las personas tienen un poco de prejuicio. Piensan «¡qué cosa!», la persona está tratando de llamar la atención de alguna manera, sobre todo cuando se trata de una lesión que no pone en peligro la vida, entonces la persona está tratando de llamar la atención.* (P1–Enfermería)

*Desgraciadamente, muchos profesionales que atienden estos casos ven al adolescente, al paciente, como alguien que quiere llamar la atención, en definitiva.* (P2–Enfermería)

Un participante mencionó la dificultad de los profesionales para ponerse en contacto con la ALNS y, a partir de ahí, tratar de comprender la experiencia de los adolescentes, pensando en la integralidad de la atención. Se comprendió la necesidad de desarrollar habilidades actitudinales que permitan entrar en contacto con el dolor del otro sin descuidar el impacto de estos fenómenos en la salud mental de los profesionales:

*Es difícil entender lo que están viviendo o pasando los adolescentes.* […] *También es un reto porque, dentro del equipo, hay muchas personas con culturas muy diferentes y autolesiones; es algo violento, impactante, que causa cierto temor en las personas* […] *esto ocurre, lo he visto. A veces he visto en equipos menos preparados para este tipo de situaciones un temor, un estigma hacia las autolesiones.* (P3–Medicina)

Las acciones ante situaciones de ALNS presentadas por los participantes pueden estar impregnadas de actitudes agresivas e imprudentes, debido al estigma presente, como describen las siguientes declaraciones:

[…] *algunas personas tienen un poco de prejuicio y terminan tratando a la persona de una manera más agresiva, pero arrogante, por saber que la persona se causó la lesión a sí misma* […] (P1–Enfermería)

*Entonces, voy a pasar esta sonda más gruesa* (nasogástrica)*; voy a buscar una vena de alto calibre con la intención de que la persona aprenda alguna lección y no intente repetir esto la próxima vez, como si el dolor que le estamos infligiendo en ese momento fuera mayor que el que se causó a sí misma*. (P1–Enfermería)

Además de estos aspectos revelados por el sentido de las ALNS, los profesionales relataron dificultades para comprender, comunicarse y cuidar a los adolescentes, lo que repercute en lo que se percibe como necesidad de salud y se ofrece como cuidado:

*Siempre es un reto, porque siempre tengo dificultades para comunicarme con este grupo*. […] *Son pacientes a los que es difícil acceder a la información, en su mayoría retraídos*. […] *es difícil acceder a la información sanitaria y al historial con estos acompañantes.* (P4–Enfermería)

*El adolescente es muy cerrado, por lo que comparte muy poco con nosotros lo que le lleva a hacer eso. Comparte muy poco de las angustias y aflicciones que le han llevado a desencadenar este tipo de comportamiento*. (P5–Enfermería)

Además, revelaron la necesidad de ampliar la mirada hacia las cuestiones psicosociales de los adolescentes atendidos, lo que conlleva la necesidad de una atención centrada en la persona, la familia y su contexto de vida. Estos aspectos dialogan fuertemente con los conceptos de atención integral:

*Las acciones dependen mucho del perfil del paciente en el momento de la consulta* […] *hay que evaluarlos individualmente para poder tomar la mejor decisión, pero es una decisión que varía mucho de un caso a otro.* (P6–Medicina)

*Hablamos mucho sobre los resentimientos que sienten al respecto, si sienten rabia, si sienten vergüenza, cómo se sienten, ¿no?* […] (P7–Medicina)

*Al tratarse de adolescentes* […] *es una situación aún más delicada porque, siempre que llegan, tenemos que ponernos en contacto con la familia y, a veces, es una familia frágil* […] (P8–Enfermería)

*También hacemos un seguimiento de los familiares del cuidador principal de ese niño, y solemos llamar a otros miembros de la familia que también conviven con él, ¿no? Con ese paciente, para investigar sus antecedentes, investigar cómo es la estructura de esa familia, cómo es el día a día de ese paciente y qué piensa la familia al respecto.* (P5–Enfermería)

Los participantes relataron situaciones en las que las ALNS pueden suponer un riesgo para la vida del adolescente y que, en ese entorno, hay que centrarse en el adolescente, estabilizar su estado y, después, comprender el significado del acto:

*Porque si ella está en la sala de urgencias, es porque ha puesto en riesgo su vida, pero no sabemos si ese riesgo fue intencional, como un intento real de suicidio, o quizá no fue intencional. Se estaba autolesionando y acabó causándose un daño que puso en riesgo su vida, así que creo que, tal vez, después de estabilizar al paciente y hablar con él, tal vez se pueda realmente diferenciar.* (P1–Enfermería)

*El primer contacto que tengo siempre es mostrar apoyo, no intentar entenderlo de inmediato.* (P1–Enfermería)

*Mis acciones consisten en intentar acoger al paciente, ¿no? Identificar también cuáles son los desencadenantes inmediatos de ese paciente y, si es posible, intentar a largo plazo.* (P3–Medicina)

Los participantes señalaron la importancia de la experiencia y la preparación profesional para sentirse seguros a la hora de prestar la atención humanizada e integral que exige este tipo de situaciones. Mencionaron la necesidad de formación continua y atención psicológica para los profesionales que trabajan allí, teniendo en cuenta la integralidad de las prácticas en el encuentro con adolescentes que se han autolesionado.

*La formación de los equipos de urgencias es esencial; se necesitan equipos capacitados para tratar a las personas de forma holística* […] (P4–Enfermería)

*Creo que la formación siempre es bienvenida, en el sentido de desmitificar y empezar a explicar realmente qué es la autolesión* […] (P7–Medicina)

*En la institución en la que trabajo, no contamos con un psicólogo que nos ayude a lidiar con situaciones como estas, ni con el usuario, el cliente, ni tampoco con nosotros, porque, en muchas ocasiones, al ser una situación delicada, también es difícil para el profesional de la salud.* (P5–Enfermería)

2. Más allá de la lesión momentánea: la organización de las prácticas y los servicios para el cuidado del adolescente que se autolesiona

Esta categoría incluyó relatos sobre la forma en que el servicio de urgencias se está organizando para atender a los ALNS en la adolescencia. En primer lugar, los participantes reiteraron que la atención humanizada y la empatía son fundamentales en la atención. Sin embargo, de acuerdo con lo que se puede ofrecer en un entorno de urgencias, que presenta características de una atención más puntual e inmediata, esta visión estuvo presente de forma más profunda entre los profesionales de enfermería:

*Cada atención debe ser individualizada; seguir protocolos no ayuda en estos casos* […] (P4–Enfermería)

*Entonces, creo que tomarle de la mano, tocarle, mirarle a los ojos, decirle que todo va a ir bien y demostrarle esa empatía, decirle que entiendo que está pasando por un momento difícil, pero que vamos a hacer todo lo posible para ayudarle, dedicarle tiempo de calidad. Entonces, tocarlo, cogerle la mano, mirarle a los ojos, permitir un momento de silencio, tal vez, para que si quiere hablar de eso en ese momento, sienta que puede tocar el tema o no. Y en ese primer momento, solo demostrar afecto y hacer que se sienta seguro y cómodo conmigo para que más tarde, tal vez, sienta que puedo tocar ese tema*. (P1–Enfermería)

Entre las limitaciones del entorno, los participantes enumeraron: el tiempo reducido de atención, señalado especialmente por los médicos; la escasez de personal multidisciplinar para prestar apoyo a los adolescentes; la falta de recursos, estructura y un lugar acogedor para la atención, señalados por el personal de enfermería, con una visión gerencial del servicio. Estos elementos estructurales y organizativos, articulados con los retos que plantea el encuentro con el adolescente que se ha autolesionado, emergen como potenciadores del sufrimiento psíquico de los profesionales:

*Siempre es un poco angustiante, es una consulta que no es rápida, que se alar*ga […] (P6–Medicina)

*Requiere una conversación, una atención que va a demandar un poco más de tiempo*. (P7–Medicina)

[…] *aquí no tenemos mucha estructura para atender estos casos psiquiátricos, ¿no? Nuestra unidad no está preparada físicamente* […] *tenemos dificultades para contactar con psiquiatría, y más concretamente con psiquiatría infantil, para que nos ayuden a llevar estos casos.* (P2–Enfermería)

*Creo que, por ejemplo, si pudiéramos contar con un equipo multidisciplinar, sería fundamental.* (P5–Enfermería)

Como potencialidad, los profesionales informaron que, en el lugar de trabajo, cuentan con la ayuda de profesionales especializados que contribuyen a mejorar la atención:

*Siempre contamos con el respaldo de psiquiatría, que es esencial para evaluar a estos pacientes* […] (P6–Medicina)

*Hay una asistente social en el servicio de urgencias, así que siempre la llamamos en este tipo de casos.* (P8–Enfermería)

Otra cuestión abordada fue la necesidad de derivar a los adolescentes a otros servicios tras el alta hospitalaria, con el fin de garantizar el proceso continuo de seguimiento de estos adolescentes con actividades de atención. Señalaron que la derivación a los dispositivos pertinentes puede ayudar a los adolescentes en ese momento, teniendo en cuenta la importancia de una transición de la atención alineada y responsable para la integralidad de las prácticas.

*Podemos derivar al paciente una vez estabilizado, después del alta, a un CAPS* […] (P1–Enfermería)

*Sí, existe la derivación* […] *pero siempre con seguimiento ambulatorio aquí en el hospital, con psiquiatría infantil* […] (P2–Enfermería)

*Y en casos más leves, también podemos derivar a la propia UBS, porque algunos tratamientos, tal vez antidepresivos o para situaciones más leves, también pueden ser tratados por el médico clínico de la UBS, pero lo ideal es que sea atendido por el CAPS, ya que ha llegado a ese nivel de autolesión.* (P1–Enfermería)

Los profesionales de enfermería mostraron gran preocupación por la transición. Los participantes mencionaron la dificultad de llevar a cabo este proceso y manifestaron el reto que supone continuar con la atención debido a la fragilidad de la red. En la mayoría de los casos, se orienta al adolescente y a la familia para que busquen atención, sin ninguna acción de coordinación o seguimiento por parte del propio servicio:

*Sé que aquí* [municipio de trabajo] *los adolescentes acaban siendo derivados al CAPSi; es una derivación difícil* […] (P7–Medicina)

*En cuanto a la derivación de la UE al CAPS, nunca lo he visto. Nunca he visto a médicos ponerse en contacto, ni a enfermeras ponerse en contacto con el CAPS más cercano para derivar al paciente y pasar el caso. Quizás haya habido orientaciones verbales, pero nada que realmente se haya hablado entre los profesionales con casos pasados y todo lo demás para el CAPS o para la propia UBS.* (P1–Enfermería)

*El adolescente sale del servicio de urgencias y emergencias con esta referencia, sale con una derivación para seguimiento psiquiátrico en el servicio de apoyo del municipio. Como nuestra red no está interconectada, no puedo decir qué desenlace tiene esa derivación al CAPS, ¿verdad? A veces, existe la posibilidad de que ese adolescente vuelva a nosotros, pero es posible que vuelva en una nueva situación puntual de automutilación o suicidio.* (P8–Enfermería)

## DISCUSIÓN

El cuidado de los adolescentes en situación de ALNS se reveló a través de prácticas impregnadas de prejuicios y estigmas, tanto hacia la ALNS como hacia la concepción de la adolescencia. Estos aspectos desafiaron la búsqueda de la integralidad en la atención, que se situó más allá de la estabilización clínica, hacia elementos psicosociales. Las acciones imprudentes y las dificultades en la comunicación, especialmente por la particularidad de «ser adolescente», hicieron que el manejo fuera más complejo, agravado por el ambiente hostil de la UE. Se hizo necesaria la educación permanente de los profesionales ante estos temas. Existe una articulación en red frágil, tan necesaria para la transición de la atención para garantizar la integralidad, a pesar de la organización propuesta por la RAUE y la RAPS.

En general, los profesionales de enfermería y medicina revelaron perspectivas similares al analizar el fenómeno. Sin embargo, se delimitaron algunas particularidades. La organización de la asistencia, el servicio y la transición de la atención fue profundizada por la enfermería. La medicina, por su parte, puso mayor énfasis en los retos de la evaluación inicial y la duración de las consultas. En este sentido, existen singularidades formativas y de actuación ante la ALNS que orientan tales perspectivas. La acogida es una práctica recomendada por la Política Nacional de Humanización, que reconoce lo que el otro aporta como una necesidad legítima y singular de salud para garantizar el acceso oportuno a las tecnologías de cuidado^(16)^. La evaluación del adolescente en situación de ALNS exige investigar sus antecedentes, experiencias previas, factores de riesgo y factores de protección, así como la intencionalidad, la letalidad y la gravedad del daño o la lesión. Ambas prácticas, la acogida y la evaluación, deben ser realizadas por todos los profesionales de la salud de nivel superior, y los cuidados deben ir seguidos de un seguimiento más específico para las diferentes categorías profesionales^(2-4)^. Aunque la enfermería cuenta con una formación que aborda más profundamente temas como la gestión de servicios y prácticas, más adelante se verá la importancia de la atención en red, organizada por todo el equipo interprofesional.

Los datos de este estudio revelaron que aún existen ideas preconcebidas y estigmatizantes sobre estos adolescentes, lo que puede desencadenar actitudes imprudentes y negligentes por parte de los profesionales de la salud. Los participantes también informaron dificultades para abordar el tema ALNS, además de los retos relacionados con la atención a la población adolescente. En esta perspectiva, un estudio brasileño realizado en el interior del estado de São Paulo con profesionales de la salud y la educación investigó sus concepciones sobre ALNS. Los resultados corroboraron los hallazgos del presente estudio: algunos profesionales mostraron incomodidad con el tema, así como comentarios que banalizaban y deslegitimaban estos comportamientos. También destacaron la ALNS como un intento de llamar la atención, articulado al proceso adolescente, sin considerar la legitimidad de estos fenómenos^(17)^.

Aun así, cuando se trata de la atención, los participantes llaman la atención sobre la importancia de una visión ampliada de las cuestiones psicosociales de estos adolescentes y de una atención centrada en las necesidades específicas de cada paciente, así como de su familia, realizada de forma humanizada. Los vínculos familiares poco saludables y sustentadores se relacionan con los comportamientos suicidas adolescentes. Aunque el alejamiento del núcleo familiar es una etapa natural del proceso de desarrollo, es fundamental que estos jóvenes cuenten con el anclaje, el apoyo y el acogimiento en el contexto familiar para hacer frente a situaciones adversas y al sufrimiento psíquico. Los profesionales que forman parte de la red de protección de la adolescencia deben orientar y apoyar a los familiares para que reconozcan su papel estratégico en el cuidado de la salud mental. Además, es imprescindible difundir información que favorezca la comprensión integral del adolescente, reforzando que su dolor debe ser validado y nunca minimizado^(18)^. Se sabe que estos aspectos son relevantes en cualquier grupo de edad, pero debido al peculiar estado de desarrollo de los adolescentes, se vuelven primordiales.

Sin embargo, existen desafíos en los sectores de la UE para lograr esta atención individualizada y humanizada al adolescente y sus familiares. Según los datos expuestos en la sección de resultados, solo la mitad de los entrevistados tuvo acceso a debates sobre ALNS en su formación. Los profesionales también señalan la necesidad de capacitar a los profesionales para que puedan actuar mejor en relación con esta demanda. Un estudio brasileño corroboró esta deficiencia, mostrando que solo un profesional había tenido contacto con el tema de la ALNS en su curso de graduación y ningún profesional había participado en formaciones durante su tiempo de servicio^(17)^. Otro estudio también reflejó la ausencia de formación formal en cursos de salud para prestar cuidados a adolescentes que ingresan en los servicios debido a situaciones de ALNS^(19)^. En este sentido, los autores destacan la importancia de construir estrategias de enseñanza y aprendizaje que contribuyan a la formación de los profesionales de la salud, así como de incluir este tema en los planes de estudio de los cursos de grado.

Se evidencia la necesidad de iniciativas destinadas a mejorar la formación de los profesionales y la preparación de los equipos de salud que trabajan en los servicios de urgencias en lo que se refiere a la salud mental, así como la capacitación continua de estos equipos. Es fundamental promover el desarrollo del conocimiento científico y sensibilizar a los profesionales que tratan directamente con esta demanda, para que comprendan que la atención en salud mental va más allá de los límites de los procedimientos técnicos, como la administración de medicamentos para aliviar el dolor. Es importante destacar que estos servicios forman parte de la RAPS, cuya función principal es la estabilización mediante cuidados iniciales. Sin embargo, estos servicios a menudo no contemplan adecuadamente las dimensiones psicosociales, lo que puede atribuirse a la laguna existente en la formación académica de los profesionales en relación con las especificidades de la salud mental^(20)^.

La perspectiva formativa de los profesionales puede influir en las dificultades para manejar el fenómeno y la población de este estudio. Se observa una falta de conocimientos, habilidades y actitudes adecuadas para actuar ante esta temática. Esta laguna se ve agravada por la propia trayectoria formativa de los profesionales, a menudo estructurada desde una perspectiva tecnicista, con un énfasis predominante en las alteraciones fisiológicas de los individuos, sin considerar, de manera amplia, los aspectos relacionados con la integralidad del ser humano. En este contexto, la educación permanente se configura como un recurso esencial para mejorar la capacitación de los equipos en el cuidado de adolescentes con sufrimiento psíquico^(21)^, incluidos aquellos en situación de ALNS.

Otros retos inherentes al entorno de la UE también se encontraron en las declaraciones de los participantes. Entre ellos se encuentran el reducido tiempo de atención, la escasez de personal multidisciplinar para prestar apoyo a los adolescentes, la falta de recursos y de una estructura adecuada para la atención, y la ausencia de un lugar acogedor para la atención. En este contexto, un estudio realizado con profesionales de la salud que trabajan en unidades de urgencias corroboró estos hallazgos, al evidenciar que la escasez de recursos humanos, equipos, materiales de consumo y la limitación de la infraestructura física constituyen barreras significativas para la efectividad de las acciones de salud^(22)^.

Esta carencia estructural y organizativa compromete la calidad de la atención prestada, interfiriendo directamente en las relaciones establecidas entre trabajadores y usuarios. Aunque existen tecnologías duras en estos entornos, no sustituyen ni reducen la importancia de las tecnologías ligeras y ligeramente duras en la atención sanitaria^(21,23)^. Las tecnologías duras se entienden como estructuras de conocimiento concretas y acabadas, como los aparatos de rayos X en los servicios de urgencias. Las tecnologías ligeras-duras son metodologías y procesos estructurados, aunque prevén la participación social y humana, como los protocolos de gestión. Por su parte, las tecnologías ligeras implican las relaciones sociales y la subjetividad incorporada en ellas^(21,23)^. En este contexto, es necesario contemplar la atención humanizada, ya mencionada, y la organización de procesos y protocolos.

En los servicios de urgencias, los profesionales ejercen sus funciones con rapidez en la toma de decisiones, guiándose por la necesidad de resolver los problemas de forma inmediata^(22)^. Además, trabajan en entornos a menudo inadecuados desde el punto de vista físico, caracterizados por la escasez de recursos y un ritmo intenso de atención, lo que los expone a importantes exigencias físicas y psíquicas. En este contexto, es esencial reconocer la importancia de la capacidad individual y colectiva para afrontar y superar los retos derivados de las diversas situaciones adversas que se viven a diario en estos espacios de trabajo. Igualmente relevante es el desarrollo de una postura institucional de atención y cuidado dirigida a los propios profesionales de la salud, que debe ser promovida por las instituciones y los líderes de los servicios^(22)^.

Los informes evidenciaron un entorno de trabajo marcado por condiciones adversas, caracterizado por la sobrecarga de tareas, la exigencia de cumplir con las actividades en poco tiempo, el número insuficiente de profesionales en los equipos, la indefinición de las funciones, la falta de respaldo institucional, las deficiencias en el espacio físico y la falta de equipos, entre otros factores. Estas condiciones inadecuadas tienden a provocar sufrimiento entre los trabajadores y comprometen la calidad de la atención prestada, una realidad que puede interferir negativamente en la oferta de una asistencia verdaderamente humanizada^(23,24)^.

Una revisión de la literatura reciente, basada en datos cualitativos, recomendó como estrategia de cuidados de enfermería en los servicios de urgencias la mejora del ambiente en las salas de espera, con el uso de kits sensoriales, auriculares y plastilina para acoger a adolescentes y jóvenes^(23)^. Estos aspectos dialogan con el concepto de servicios y prácticas amigables para los adolescentes, que se relacionan con las necesidades de información, alfabetización en salud, accesibilidad, equidad y seguridad en los contextos de atención. La construcción de relaciones de confianza y comodidad también se consideran esenciales para los adolescentes^(25)^. Estos elementos se articulan con la Acción Global Acelerada para la Salud de los Adolescentes, que señala la importancia de priorizar a esta población en las inversiones y la participación en salud^(26)^.

Otro aspecto relacionado con lo anterior y que fue señalado por los profesionales de este estudio es el que se refiere a la atención de la salud mental de los profesionales de la salud que trabajan en este ámbito. Se sabe que el equipo de salud que trabaja en los servicios de urgencias puede ver mermada su calidad de vida, ya que se enfrenta a diario a una sobrecarga de trabajo con un horario agotador, escasez de recursos humanos y falta de reconocimiento profesional. Además, las UE se consideran entornos estresantes, con características como la imprevisibilidad y el alto flujo de atendidos, lo que exige capacidad de organización y toma de decisiones en equipo^(23,27,28)^. Otras características de estas unidades que pueden aumentar el estrés laboral son el ruido, la falta de materiales, la reducción del número de profesionales, la jornada doble y la baja remuneración^(23,27,28)^.

Además de los problemas y dificultades a los que ya se enfrentan los enfermeros en las UE, el tema de las autolesiones y el suicidio puede provocar en los profesionales sentimientos de preocupación, impotencia, ansiedad, angustia, tristeza, frustración, indignación, pérdida y miedo^(29)^. Aquí se tendrá en cuenta la gran dificultad de diferenciar la ALNS y el intento de suicidio tan pronto como el paciente llega al servicio, tal y como señalaron los propios participantes en la investigación, ya que es necesario, en un primer momento, estabilizar al paciente para, a continuación, realizar el trabajo de investigación, intervención y prevención de nuevas situaciones. En este sentido, un estudio coreano^(30)^ analizó el agotamiento emocional de los profesionales de enfermería que trabajan en la prevención del suicidio en los servicios de salud mental. Según los autores, este agotamiento estaba relacionado con algunos factores. Entre ellos se encontraban la falta de confianza en sus habilidades y la percepción de sus límites en la capacidad de respuesta ante situaciones de crisis. En relación con esto último, informaron de su insatisfacción por la sensación de estar siempre haciendo lo mínimo y de no tener tiempo ni estructura para hacer frente a situaciones de crisis debido a la gran sobrecarga de trabajo. También aparecieron factores como: sentirse impotente y frustrado por no poder ayudar al paciente; tener una fuerte empatía e identificación excesiva con el paciente, compartiendo los sentimientos de tristeza y desesperación con la persona atendida; dificultad para comprender al paciente, oponiéndose al suicidio en sus valores personales; y creer que los pensamientos suicidas de las personas que lo intentan no mejoran^(30)^.

Se percibe que existe un sufrimiento psicológico y emocional en los profesionales que trabajan en los sectores de la UE y que tienen, entre sus demandas, aquellas relacionadas con la salud mental, ALNS e intentos de suicidio, especialmente cuando se trata de adolescentes. Por lo tanto, algunos autores proponen posibilidades de atención en salud mental para estos trabajadores. Algunas de las iniciativas son la gimnasia laboral y el monitoreo de la salud mental de los profesionales, estimulando estrategias individuales como el ocio con ejercicio físico, música, terapia, caminatas, además de acompañamiento psicológico^(23,27,28)^. También es necesario mejorar la remuneración y valorar al profesional^(27)^. Otras recomendaciones para mejorar la atención de la ALNS en la adolescencia son: estructurar el entorno de trabajo para que los profesionales tengan tiempo suficiente para realizar las intervenciones; promover programas educativos que mejoren sus habilidades de intervención; promover programas educativos que les ayuden a mantener una distancia terapéutica con el paciente y a regular y gestionar sus propias emociones, evitando una implicación excesiva en situaciones desesperadas y el sentimiento de tristeza; comprender a los pacientes más desde el punto de vista psicológico que moral; y ayudarles a lidiar con sus propias emociones^(30)^.

Por último, en este estudio surgieron las debilidades para el trabajo en red, teniendo en cuenta la integralidad de las prácticas. De este modo, es posible, por ejemplo, realizar un trabajo de articulación y matriculación para identificar los casos que necesitan atención individual y especializada, y aquellos que pueden beneficiarse de trabajos educativos y multiprofesionales y otras iniciativas de atención en salud mental. Para ello, se puede contar con los dispositivos que se encuentran en las RAPS^(31)^.

Las RAPS cuentan con servicios de atención básica, especializada, hospitalaria, de urgencias y otros servicios de acogida transitoria, estrategias de desinstitucionalización y rehabilitación psicosocial^(11)^. La unidad de urgencias, por lo tanto, cuenta con una red de servicios que pueden articularse y activarse para la atención en salud mental, incluso en situaciones de ALNS. Así, es importante articular un trabajo conjunto con los diferentes servicios disponibles en la red de cada municipio, con el fin de desarrollar cuidados y acciones coordinadas^(32)^, a partir, por ejemplo, de la comunicación efectiva entre los servicios y la elaboración de Planes Terapéuticos Singulares para los usuarios de la RAPS^(31)^. En este ámbito, es esencial producir prácticas y servicios amigables para los adolescentes en otros puntos de atención.

La literatura presenta algunas iniciativas que hacen posible esta articulación entre instituciones para la atención en salud mental de los usuarios de la red. Entre ellas, se encuentran estudios de casos conjuntos, reuniones intersectoriales sistemáticas, atención compartida y acciones de apoyo matricial. La articulación intersectorial, con la participación de instituciones de otros sectores, como la asistencia social y la educación, se señala como necesaria para la integralidad de la atención^(32)^. Esto propicia una visión global y acciones dirigidas a las individualidades y singularidades que demandan los cuidados en salud mental de los usuarios de la RAPS.

Como limitaciones, el estudio solo incluye la investigación con profesionales de las áreas de enfermería y medicina. Hoy en día, es posible encontrar diversos profesionales de la salud que actúan activamente en los sectores de la UE, ya sea como parte del equipo fijo del entorno o como equipo de apoyo a partir de interconsultas. De este modo, otras categorías profesionales, como asistentes sociales, psicólogos o nutricionistas, podrían haber sido incluidas entre los profesionales entrevistados, lo que habría garantizado una visión aún más amplia de las situaciones y los pacientes. Además, habría sido interesante contar con la opinión de profesionales de nivel técnico y medio, como los del área de enfermería y recepción.

A pesar de las limitaciones, este estudio avanza en el área de la salud mental de los adolescentes en situación de ALNS, al identificar los retos presentes en un servicio poco estudiado y articulado con esta población. Estos retos pueden convertirse en áreas clave para las intervenciones, como el cuidado del entorno y la acogida/evaluación de las situaciones vividas. También identifica la importancia de pensar en flujos y protocolos que fortalezcan y subsidien la transición de la atención de los adolescentes desde el SUH. De este modo, es necesario que futuros estudios profundicen en las posibles contribuciones al área de la enfermería en el papel de gestión y articulación de prácticas y servicios para la salud de los adolescentes.

## CONCLUSIÓN

El concepto de integralidad permitió comprender tanto la atención en salud dirigida a adolescentes en situación de ALNS en servicios de UE, como la organización de los servicios. Existen importantes desafíos relacionados con la forma de trabajar en estos escenarios y fragilidades formativas en los temas de salud mental y adolescencia. La articulación en red se ve dificultada por este modelo de atención, a menudo basado en la lógica del derivamiento.

Se necesitan nuevas formas de producción de salud para los adolescentes orientadas a las demandas actuales de atención. Este aspecto abarca desde la formación, mediante estrategias activas y participativas a partir de contextos sociales y epidemiológicos, hasta una gestión de la atención y los servicios basada realmente en la integralidad. Se recomiendan nuevos estudios, especialmente en el Sur Global, orientados a escuchar a otros profesionales y a los propios adolescentes.

## Data Availability

Los contenidos subyacentes al texto de la investigación están contenidos en el manuscrito.
